# Infection experiments with novel *Piscine orthoreovirus* from rainbow trout (*Oncorhynchus mykiss*) in salmonids

**DOI:** 10.1371/journal.pone.0180293

**Published:** 2017-07-05

**Authors:** Helena Hauge, Niccolo Vendramin, Torunn Taksdal, Anne Berit Olsen, Øystein Wessel, Susie Sommer Mikkelsen, Anna Luiza Farias Alencar, Niels Jørgen Olesen, Maria Krudtaa Dahle

**Affiliations:** 1 Norwegian Veterinary Institute, Oslo & Bergen, Norway; 2 National Veterinary Institute, Technical University of Denmark, Copenhagen, Denmark; 3 Department of Food Safety and Infection Biology, Norwegian University of Life Sciences, Oslo, Norway; James Cook University, AUSTRALIA

## Abstract

A new disease in farmed rainbow trout (*Onchorhyncus mykiss*) was described in Norway in 2013. The disease mainly affected the heart and resembled heart and skeletal muscle inflammation (HSMI) in Atlantic salmon (*Salmo salar* L.). HSMI is associated with *Piscine orthoreovirus* (PRV), and a search for a similar virus in the diseased rainbow trout led to detection of a sequence with 85% similarity to PRV. This finding called for a targeted effort to assess the risk the new PRV-variant pose on farmed rainbow trout and Atlantic salmon by studying infection and disease pathogenesis, aiming to provide more diagnostic knowledge. Based on the genetic relationship to PRV, the novel virus is referred to as PRV-*Oncorhynchus mykiss* (PRV-*Om)* in contrast to PRV-*Salmo salar* (PRV-*Ss)*. In experimental trials, intraperitoneally injected PRV*-Om* was shown to replicate in blood in both salmonid species, but more effectively in rainbow trout. In rainbow trout, the virus levels peaked in blood and heart of cohabitants 6 weeks post challenge, along with increased expression of antiviral genes (Mx and viperin) in the spleen, with 80–100% of the cohabitants infected. Heart inflammation was diagnosed in all cohabitants examined 8 weeks post challenge. In contrast, less than 50% of the Atlantic salmon cohabitants were infected between 8 and 16 weeks post challenge and the antiviral response in these fish was very low. From 12 weeks post challenge and onwards, mild focal myocarditis was demonstrated in a few virus-positive salmon. In conclusion, PRV-*Om* infects both salmonid species, but faster transmission, more notable antiviral response and more prominent heart pathology were observed in rainbow trout.

## Introduction

Rainbow trout (*Onchorynchus mykiss*) farming is common in many parts of the world. This salmonid is highly domesticated, fast-growing, and an attractive food source. In 2015, European production of rainbow trout was estimated to be 386 thousand tons, making it the second most farmed fish species in Europe after Atlantic salmon (*Salmo salar* L.), with 1.57 million tons of salmon produced during the same period [1]. Rainbow trout are primarily produced as portion-sized fish in fresh water and river raceways, whereas larger rainbow trout is primarily sea-reared on the north European coast together with Atlantic salmon.

Viral diseases pose a constant challenge for the sustainable development of aquaculture. In 2013, a new disease was diagnosed in fresh water hatcheries of rainbow trout, 25–100 g, in Norway [2]. Mortalities were moderate to high, and the disease was also observed after sea water transfer. The main pathological findings were signs of circulatory failure, inflammation of the heart and red skeletal muscle and necrosis in the liver, resembling the pathology described for heart and skeletal muscle inflammation (HSMI) in Atlantic salmon [3,4]. In addition, the rainbow trout suffered from anaemia, which is not common for HSMI. HSMI in Atlantic salmon is associated with *Piscine orthoreovirus* (PRV), a non-enveloped virus with a segmented genome of double-stranded RNA [5,6]. PRV are found ubiquitously in farmed salmon, and are not always associated with clinical disease or histopathological changes resembling HSMI [7,8]. Samples from the diseased rainbow trout were negative in PRV RT-qPCR assays, but by applying several assays targeting different PRV-segments followed by Sanger sequencing, a gene sequence with 85% identity to parts of PRV-segment S1 was obtained [2]. High levels of the new PRV-variant were detected in diseased rainbow trout from all affected farms, strongly suggesting that this was the etiological agent of the disease.

PRV has previously been shown to infect circulating red blood cells prior to infection of cardiomyocytes in Atlantic salmon [9,10]. Although the highest levels of virus is detected in blood [10], the blood cell infection does not lead to any obvious anaemia in Atlantic salmon challenge studies. This is in contrast to the anaemia associated with the new PRV*-*variant in rainbow trout outbreaks [2].

The finding of a putative new emerging viral disease in rainbow trout in Norway is of significant concern for the industry worldwide, making it necessary to assess its significance in salmonids and the risk if the virus spreading to other rainbow trout producing countries. Furthermore, several viral diseases have been found to transmit between Atlantic salmon and rainbow trout [11,12]. Since the disease outbreaks occurred in Norway where Atlantic salmon is farmed intensively, and in some areas in the same fiords as farmed rainbow trout, investigating if Atlantic salmon are susceptible to the virus is also relevant. In the present article the novel PRV-variant is referred to as PRV-*Om* (PRV from *Onchorhynchus mykiss*), awaiting the official nomenclature. The corresponding PRV from Atlantic salmon is referred to here as PRV-*Ss* (PRV from *Salmo salar* L.*)*.

We present results from infection experiments with PRV-*Om* in rainbow trout and Atlantic salmon, respectively. Experiments were performed to reveal the ability of the virus to infect the two salmonid species. The aim was also to determine whether the virus is associated with the new disease in rainbow trout, and whether it may also induce disease in Atlantic salmon.

## Materials and methods

### Challenge experiments

A total of three challenge experiments were performed; a small scale preliminary study including both rainbow trout and Atlantic salmon (trial 1A and B) and two separate long-term studies in rainbow trout (trial 2) and Atlantic salmon (trial 3), respectively (Table 1).

**Table 1:** PRV-*Om* challenge trials, overview of samples and analyses.

			Duration (weeks)		Sampling, weeks post challenge (WPC)		Number of fish sampled, injected+cohabitants	Samples from	Analyses
**Trial 1**	**Rainbow trout and Atlantic salmon**								
1A	Tank 1	20 virus injected		8		0, 1, 2, 4, 6, 8	4+4^1^^,^^2^	Blood	RT-qPCR*
								Spleen	RT-qPCR
	Rainbow trout							Heart	RT-qPCR; histology^3^
		20 cohabitants						Head kidney	RT-qPCR
1B	Tank 2	20 virus injected		8		0, 1, 2, 4, 6, 8	4+4^1^^,^^2^	Blood	RT-qPCRR
								Spleen	RT-qPC
	Atlantic salmon							Heart^2^	RT-qPCR; histology^3^
		20 cohabitants						Head kidney	RT-qPCR
**Trial 2**	**Rainbow trout**								
	Tank 1	40 virus injected		4		1, 2, 3	5	Blood	RT-qPCR
		“Early stage infection”							
	Tank 2	30 virus injected		14		4, 8, 12	5+5	Blood	RT-qPCR; IF**
								Spleen	viperin, Mx*** RT-qPCR
		30 cohabitants						Heart	RT-qPCR; histology
	Tank 3	30 virus injected		14		6, 10, 14	5+5	Blood	RT-qPCR; IF
								Spleen	viperin, Mx RT-qPCR
		30 cohabitants						Heart	RT-qPCR; histology
	Tank 4	40 mock injected		14		4, 10, 12, 14	5+5^4^	Blood	RT-qPCR; IF
								Spleen	viperin, Mx RT-qPCR
		40 cohabitants						Heart	RT-qPCR; histology
**Trial 3**	**Atlantic salmon**								
	Tank 1	90 virus injected		16		4, 6, 8, 10, 12, 14, 16	8+8	Blood	RT-qPCR; IF, Hgb****
		90 cohabitants						Spleen	viperin, Mx RT-qPCR
								Heart	RT-qPCR; histology
	Tank 2	50 non-infected fish		16		4, 6, 8, 10, 12, 14, 16	4	Blood	RT-qPCR; IF
								Spleen	viperin, Mx RT-qPCR
								Heart	RT-qPCR; histology

^1^ **Trial 1,** samples for RT-qPCR–exceptions from 4+4. 1A: 4 WPC: 4 injected + 3 cohabitants, 8 WPC: 3 injected + 5 cohabitants. 1B: 6 WPC: 3 injected + 3 cohabitants, 8 WPC: 2 injected + 4 cohabitants

^2^ **Trial 2,** samples for histology—exceptions from 4+4. 1A 8 WPC: 3 injected + 6 cohabitants, 1B: 6 WPC: 3 injected + 3 cohabitants, 8 WPC: 2 injected + 5 cohabitants

^3^ **Trial 1,** 1A and 1B: Additional organs for histology: liver, kidney, spleen, exocrine pancreas, pyloric caeca, red and white skeletal muscle

^4^ **Trial 2**: Samples for histology: 4 WPC: 3 injected + 3 cohabitants, 10 and 12 WPC: 5 injected only. 14 WPC: 3 injected + 2 cohabitants

^*^ RT-qPCR = RT-qPCR for PRV-*Om*

** IF = Immunofluorescence immunostaining for PRV-*Om*

*** Mx = Myxovirus resistance protein

**** Hgb = haemoglobin

The experimental studies performed in Norway (trials 1 and 3) were carried out in accordance with the recommendations in the current animal welfare regulations: FOR-1996-01-15-23 (Norway), and the protocols were approved by the Norwegian Animal Research Authority. Trial 1 was performed in the research aquarium facility at the Norwegian Veterinary Institute (NVI), and the long-term study in Atlantic salmon (trial 3) was performed at the VESO Vikan aquatic research facility in Namsos, Norway. Trial 2 was carried out in the experimental facilities at DTU-VET in Denmark in accordance with the recommendations in the current animal welfare regulations under the licence 2013-15-2934-00976. The protocols were approved by the Danish Animal Research Authority. The health status and environmental conditions of the fish were monitored daily.

#### Rainbow trout and Atlantic salmon study (trials 1A and B)

Rainbow trout, 100–200 g (n = 44) (AquaGen origin), and Atlantic salmon pre-smolts (indigenous to the river Drammenselva, Buskerud County, Norway) with a mean weight of 40 g (n = 44) hatched at the Hellefoss cultivation station, were transported to the research aquarium facility at the NVI and acclimatized for two weeks. The fish were reared in standard 160 L (Atlantic salmon) or 500 L (rainbow trout) fibreglass tanks at comparable stocking density of 11–13 g/L with flow-through fresh water supplied from the municipal water works. The water was passed through a carbon filter column and aerated mechanically in the aquarium facility, before entering the fish tanks. Water temperature and oxygen saturation were monitored daily and ranges were 10.5 ± 1,2°C, 12:12 light:darkness (L:D) cycle and > 80% O_2_-saturation, during the study period. The fish were hand-fed a 2 mm pelleted commercial diet (Skretting, Stavanger, Norway) at a rate of 2% of calculated biomass/tank/day.

The preparation of the challenge material was based on HSMI challenge experiments previously performed in Atlantic salmon using blood as the source of virus [10].

The challenge inoculum was prepared from blood cell pellets collected from three individual fish from a hatchery outbreak in rainbow trout in January 2014 (Hatchery D) [2], with a blood level of PRV-*Om* corresponding to a mean Ct value of 19.5. Fish from this outbreak were screened for PRV-Ss, infectious salmon anaemia virus (ISAV), salmonid alphavirus (SAV), piscine myocarditis virus (PMCV), viral haemorrhagic septicaemia virus (VHSV), infectious hematopoietic necrosis virus (IHNV) and infectious pancreatic necrosis virus (IPNV) with negative results (2). The blood was centrifuged (850 x g, 4°C, 10 min), plasma was removed, and the red blood cell pellets were stored at -80°C. For preparation of challenge inoculum, the pooled blood cell pellet was diluted 1:3 in Leibovitz’s L-15 medium (ThermoFisher Scientific Inc, USA), freeze-thawed in three rapid cycles, and then diluted further to a 1:10 ratio in L-15 medium.

Prior to the initiation of the experiment, the Atlantic salmon were tested for PRV-*Ss*, SAV, PMCV, and IPNV, and the rainbow trout for PRV-*Om* by RT-qPCR using primers and probes as described elsewhere [2,7,13–15]. Four fish of each species were also sampled as negative controls. Fifty percent of the remaining fish (n = 20) were anesthetized in water containing benzocaine (80 mg/L), marked by clipping of the adipose fin, and intraperitoneally (i.p.) injected with 0.1 ml of the diluted challenge inoculum (shedders) before transfer to a tank containing an equal number of naïve cohabitants (n = 20). Four injected fish and four cohabitants from each group were then sampled at 1, 2, 4, 6 and 8 weeks post challenge (WPC) (see Table 1 for overview).

#### Rainbow trout long-term study (trial 2)

Rainbow trout were obtained from eyed eggs provided by a Danish commercial fish farm officially registered free of IPNV, IHNV, VHSV and bacterial kidney disease (BKD). After disinfection procedures with Iodine, the fish eggs were hatched, and fish were grown in the wet laboratory facilities of the European Union Reference Laboratory for fish disease (EURL, Copenhagen, Denmark) in tap water recirculated and disinfected by UV light. Before infection, the specific pathogen free (SPF) rainbow trout were moved into the high containment infection facility with flow-through fresh water system with constant temperature of 12°C ±1°C.

For production of challenge material, fish (n = 15) with an average weight of 380 g were anesthetized in water containing benzocaine (80 mg/L, Sigma) and i.p. injected with 0.1 ml of infected blood cell pellet diluted 1:4 v/v in L-15 medium. The challenge material injected was obtained from injected rainbow trout from trial 1, consisting of blood cells sampled 4 and 6 WPC (mean Ct value 25). Every week, starting 1 WPC, five fish were anesthetized and 1 ml of blood was collected into heparin-coated tubes (BD Vacutainer^®^) from the caudal vein and immediately stored cold for analysis of PRV-*Om* levels. These fish were marked by clipping of the adipose fin to avoid multiple sampling of the same fish during the experiment, and re-introduced to the tank. At 3 WPC one fish, previously sampled for blood, was found dead in the experimental tank, and PRV-*Om* RNA was detected in heart, spleen and kidney (Ct values of 29.0, 26.3 and 29.2, respectively). At this time point, nine fish were euthanized with an overdose of anaesthesia and bled, while the remaining five fish were euthanized and bled 4 WPC. During the four weeks, the PRV-*Om* analyses indicated an active replication of virus, similar to the observations in trial 1. Two out of five fish were positive for the virus at 1 and 2 WPC, 4 out of 5 at 3 WPC, and 5 out of 5 at 4 WPC. Virus positive blood samples collected from the nine individuals sampled at 3 and 4 WPC (Ct-range 18.4 to 26.2), were pooled and stored at -80°C for use as inoculum.

For the long-term rainbow trout trial (Trial 2), 240 SPF rainbow trout of 32 g in average were divided into four 150 L tanks (Table 1) run with 15 L/h flow-through fresh water renewal at the following conditions: 12°C±1°C, L:D 12:12, stocking density below 60 kg/m^3^, and feeding of 1.5% of biomass.

Fish were anaesthetized prior to the infection by immersion in water containing benzocaine (80 mg/L water) and then i.p. injected with 0.1 ml of challenge inoculum (pooled infected rainbow trout blood 1:4 v/v diluted in L-15 medium). Mock infection with naïve blood diluted in L-15 medium was performed in the same manner on 50% of the fish in tank 4 (non-exposed control group). Injected fish were marked by adipose fin clipping in tanks 2–4. In tank 1, defined as “early stage infection”, five fish were sampled and their blood tested at 1, 2, and 3 WPC to monitor the early infection kinetics in i.p injected fish. In tank 2 and 3 five injected fish and five cohabitants were sampled at 4, 6, 8, 10, 12 and 14 WPC from alternate tanks. In tank 4 (non-exposed control), five mock injected fish and five cohabitants were sampled at 4, 10, 12 and 14 WPC (see Table 1 for overview).

Also in this study, one injected fish was found dead 25 days post challenge. Bacteriological analysis was performed according to standard diagnostic procedures, by streaking kidney tissue onto blood agar (BA) and Tryptone Yeast Extract Salts (TYES), followed by incubation for one week at 20°C and 15°C, respectively.

#### Atlantic salmon long-term study (trial 3)

For the long-term challenge experiment in Atlantic salmon, parr of the SalmoBreed standard strain (n = 238, 30–40 g) were transferred from the VESO Vikan hatchery to the research facility and acclimatized for two weeks. During the experiment the fish were reared in flow-through tanks in fresh water at the following conditions: 12°C±1°C, L:D 24:0, stocking density below 80 kg/m^2^ and feeding of 1–2% of biomass.

Challenge material was prepared from mixed blood cell pellets taken from the peak viraemic phase in rainbow trout shedders (4 and 6 WPC) from trial 1A. Virus levels in the material ranged from Ct 16 (4 WPC) to Ct 19.5 (6 WPC) as measured in whole blood prior to freezing and thawing. At the day of challenge blood cell pellets corresponding to 10% of the challenge inoculate volume were pooled, diluted 1:3 in L-15 medium and sonicated on ice (8 x 10 sec pulses at 25 Hz with 30 sec rest in between), followed by centrifugation (3000 rpm, 4°C, 10 min) to remove cellular debris, after which the material was added sterile PBS to a final 6-fold dilution. Fish dedicated to be shedders (n = 90) were injected i.p. with 0.1 ml of challenge material under anaesthesia with benzocaine (80 mg/L) and marked by cutting of the adipose fin. An equal number of naïve cohabitants (n = 90) were added to the shedder tank. Fifty naïve fish were kept in a separate tank as controls. From 4 WPC and every second week until 16 WPC, eight shedders, eight cohabitants and four control fish were sampled (see Table 1 for overview).

#### Stress test of Atlantic salmon cohabitants

At 10 WPC in the Atlantic salmon long-term study, a stress test was performed on cohabitant fish and controls. Thirty cohabitants from tank 1 and fifteen non-exposed control fish were labelled by clipping (cohabitants in the left maxilla, controls in the fat fin) and transferred to two separate containers, each equipped with controllable air flow (20% oxygen) and oxygen monitoring equipment. The containers had a diameter of 70 cm, and the fish density was approximately 50 kg/m^3^. The initial oxygen level in the water was 80% and the temperature 12°C. The reduction to <50% oxygen was performed by shutting down the air flow for approximately 15 min. The oxygen level was then kept between 40 and 50%, constantly monitored and manually regulated for 4 h, after which the oxygen level was increased to >70%. The fish were then returned to their original tanks. At 12, 14 and 16 WPC the stress tested fish (eight cohabitants and four controls) were sampled.

#### Sampling

Upon sampling, fish were euthanized with an overdose of benzocaine (800 mg/L), measured and weighed. Blood was collected from the caudal vein on heparinized tubes (BD Vacutainer ®, New Jersey, USA) for RT-qPCR analysis and blood smears prepared for immunofluorescence staining of blood cells. Tissue samples were collected for histological analyses (see *Histology* for details) and tissue samples in RNALater® (ThermoFisher Scientific Inc, USA) for RT-qPCR analyses as specified in Table 1.

### Haematocrit and haemoglobin assays

In study 3 with Atlantic salmon, haematocrit (HCT) and haemoglobin (HGB) levels were measured in blood. HCT was measured using standard procedures with micro haematocrit tubes (GMBH) and centrifugation directly after blood sampling. HGB was measured using a HemoCue Hb 201+ (HemoCue AB, Ängelholm, Sweden) with corresponding cuvettes, performed according to the manufacturer’s protocol.

### Detection of viral RNA by RT-qPCR

For trials 1 and 3, 50 μL of whole blood was transferred to NucliSENS easyMag lysis buffer (280134, bioMérieux, Marcy l’Etoile, France) before isolation of nucleic acids (easyMag, bioMérieux). Total RNA from heart tissue was isolated using QIAsymphony SP and the RNeasy Mini Kit (QIAGEN, Hilden, Germany) according to the manufacturer´s recommendations. The RNA-concentration was measured using a NanoDropTM 2000 spectrophotometer (Thermo Scientific, Wilmington, DE, USA), and the samples were stored in RNase-free water at -80°C. RT-qPCR was performed on 500–1000 ng total RNA using the OneStep RT-PCR kit (QIAGEN), primers, probes and conditions as described elsewhere [2] and according to the manufacturer’s instructions for the kit.

For rainbow trout in trial 2, RT-qPCR was performed on RNA purified from blood cells after centrifugation of blood for 10 min at 1000×g, plasma removal and dilution of cells in 1:4 proportions in L-15 medium. RNA was purified using the Qiagen RNA Mini Blood Kit (QIAGEN).

All RT-qPCR analyses were performed using Agilent Mx3005P and Mx3000P qPCR-system (Agilent Technologies, Santa Clara, United States), and MxPro (v. 4.10) software. Baseline and cycle threshold (Ct) were set manually.

### Histology

According to Olsen et al. 2015 [2], the heart was the organ most consistently affected in diseased rainbow trout in field cases. Accordingly, the histological examination included hearts from all fish in all trials, and in addition red and white skeletal muscle and liver for a selection of the individuals. In the experiments 1A and 1B, kidney, spleen, exocrine pancreas, pyloric caeca, and at 4 WPC gills, were also examined (S1 Data file).

In trial 1, histological examination was performed from 4 to 8 WPC on tissues from 25 rainbow trout (trial 1A) and 17 Atlantic salmon (trial 1B) (Table 1). Three non-exposed fish of each species were also included. In trial 2, hearts from 60 exposed and 21 non-exposed rainbow trout and muscle and liver from 23 individuals, 19 exposed and 4 controls were examined by histology. In trial 3, hearts from 104 exposed and 40 non-exposed Atlantic salmon were examined. See Table 1 for details.

Tissue samples collected in 10% neutral buffered formalin were embedded in paraffin and processed into sections of 3–4 μm, stained with haematoxylin and eosin (H&E) and examined by light microscopy. Pathological findings in the heart were scored as mild (one to a few lesions), moderate (more extended distribution of lesions) and severe (most of the heart sample affected).

### Immunofluorescence staining of blood cells

Selected blood samples from the long-term challenge experiments in rainbow trout (trial 2) and Atlantic salmon (trial 3) were stained for viral protein in blood cells. In both trials a polyclonal rabbit antiserum against PRV-*Ss* putative outer capsid protein σ1 (Anti-σ1, #K275 [10]) was used.

For rainbow trout, the panel of samples represented eight fish, including two samples from negative control fish, one virus-negative cohabitant and five virus-positive cohabitant fish at the peak of the infection (6 WPC). Previously obtained results from PRV*-Ss* in Atlantic salmon from Finstad et al 2014 [10] and Wessel et al 2015 [16] were used as positive controls.

Blood smears were air dried and fixated with ice cold methanol (Emsure ®) for 5 min. Samples were blocked (1.5% BSA in 1 x PBS with 0.05% Tween 20 and 150 mM NaCl) for 30 min at 37°C, and stained with anti-σ1 (1:1000) and secondary antibody, isothiocyanate-fluoresceinated mouse anti-rabbit Ig (DAKO), for 30 min at 37°C, respectively. Samples were washed with PBS four times between each step. The slides were rinsed in water, air-dried, and mounted in Vectashield (Vector Laboratories Inc., Burlingame, CA) for epifluorescence microscopy. An Axioimager M1 epifluorescence microscope equipped for epifluorescence with a 100-W HBO lamp and filter sets 43 and 38 was used to visualize Cy3 and FITC, respectively. Images were obtained using an AxioCam MRm version 3 FireWiremonocrome camera and AxioVision software, version 4.5 (Carl Zeiss, Oberkochen, Germany).

For the Atlantic salmon analysis 10 μL heparinized blood from fish C 12-1-5 (S1 Data file, Ct value of 18.9) was diluted 1:50 in staining buffer (PBS + 1% BSA + 0.05% azide) and plated into 96-well plates (75 μL per well). All incubations were performed on ice. The cells were washed in staining buffer, fixed in IC fixation buffer (eBioscience, San Diego, CA, USA) for 20 min and washed with Permeabilization buffer (eBioScience). Cells were stained with anti-PRV σ1 (1:5000) for 30 min and secondary Alexa Fluor 488 conjugated anti-rabbit IgG (Molecular Probes, Eugene, Oregon, USA) (2 mg/ml diluted 1:800) for 30 min using Permeabilization buffer in all washes and dilutions. Finally, the nuclei were stained with Hoechst 33342 (Invitrogen) before the cells were transferred to Countess chamber slides (Invitrogen) and inspected by immunofluorescence microscopy. Images were captured on an inverted fluorescence microscope (Olympus IX81).

Staining of double stranded RNA (S1 Fig) was performed on blood smears from a rainbow trout sample from Hatchery D [2], also used to prepare the initial challenge material. In short, blood smears were air dried before fixation in ice cold methanol. Slides were then rehydrated in PBS and blocked in PBS with 5% skimmed milk before staining with monoclonal antibody against dsRNA (J2; 1:200) (Scicons, Hungary), washed and stained with secondary antibody; goat anti-mouse Alexa Fluor 595 (1:1000) from Molecular Probes (Life Technologies). The nuclei were stained with Hoechst 33342 (Life technologies). Coverslips were mounted with Fluoroshield (Sigma-Aldrich) and visualized on an inverted fluorescence microscope (Olympus IX81).

### Immunohistochemistry heart

Sections from formalin-fixed and paraffin-embedded heart samples were examined for PRV-*Om* by immunohistochemistry using rabbit antiserum against the PRV-*Ss* capsid proteins σ1 (#K275) and μ1c (#K265) [9]. Parallel sections were also examined using antibodies against dsRNA (J2; 1:800) (Scicons, Hungary) [17] using the same protocol as for the PRV antisera. Heart samples from five rainbow trout (trial 2) and five Atlantic salmon (trial 3) with myocarditis and low PRV-*Om* Ct values in blood and/or heart were examined.

### Antiviral gene expression analysis

Spleen samples from rainbow trout cohabitants from trial 2 (n = 5 per sample point) and Atlantic salmon cohabitants from trial 3 (n = 8 per sample point) were homogenized individually in 500 μL QIAzol Lysis Reagent (QIAGEN) with 5 mm steel beads and TissueLyser II (QIAGEN). Total RNA was isolated by chloroform extraction and ethanol precipitation and loaded onto an RNeasy mini spin column (QIAGEN). Further purification was according to the RNeasy kit instructions, and the final RNA concentration was measured using a NanoDropTM 2000 spectrophotometer. Total RNA (500 ng) from each sample was used for cDNA synthesis using the QuantiTect Reverse Transcription kit (QIAGEN) with a genomic DNA elimination step. For RT-qPCR analysis, cDNA corresponding to 10 ng RNA was analysed in triplets for 40 cycles of 94°C/15 sec, 60°C/30 sec. Levels of elongation factor 1 α (EF1α), Mx-1, and viperin-1 mRNA were assessed in both species using 500 nM primers and the Maxima SYBR Green/ROX qPCR Master Mix (Fisher scientific). Primers used are previously published [18,19]. The specificity of the SYBR Green assay was confirmed by melting point analysis. An eight point concentration standard curve (two-fold dilutions) made from a representable mix of samples was run on each plate and used to calculate relative gene expression differences. Levels of EF1α mRNA [20] were used for normalization.

### Statistical analysis

A Chi-square test with the confidence limit set at 95% (p<0.05) was used to verify the group association between positive PRV-*Om* RT-qPCR results in blood and the histopathological findings in heart for both long-term trials (Trial 2 and 3, S1 Table). For the immune response data, a one-way ANOVA with Dunnett’s multiple comparison test was used to calculate significant differences from control fish. For correlating hematocrit and hemoglobin levels, a Pearson r correlation was performed.

## Results

### Trial 1, rainbow trout and Atlantic salmon

An 8 week study including both rainbow trout and Atlantic salmon (trial 1A and 1B, respectively, Table 1) was performed to see if an infection could be established in these species and if virus could be transmitted to naïve cohabitants.

**Fig 1:**
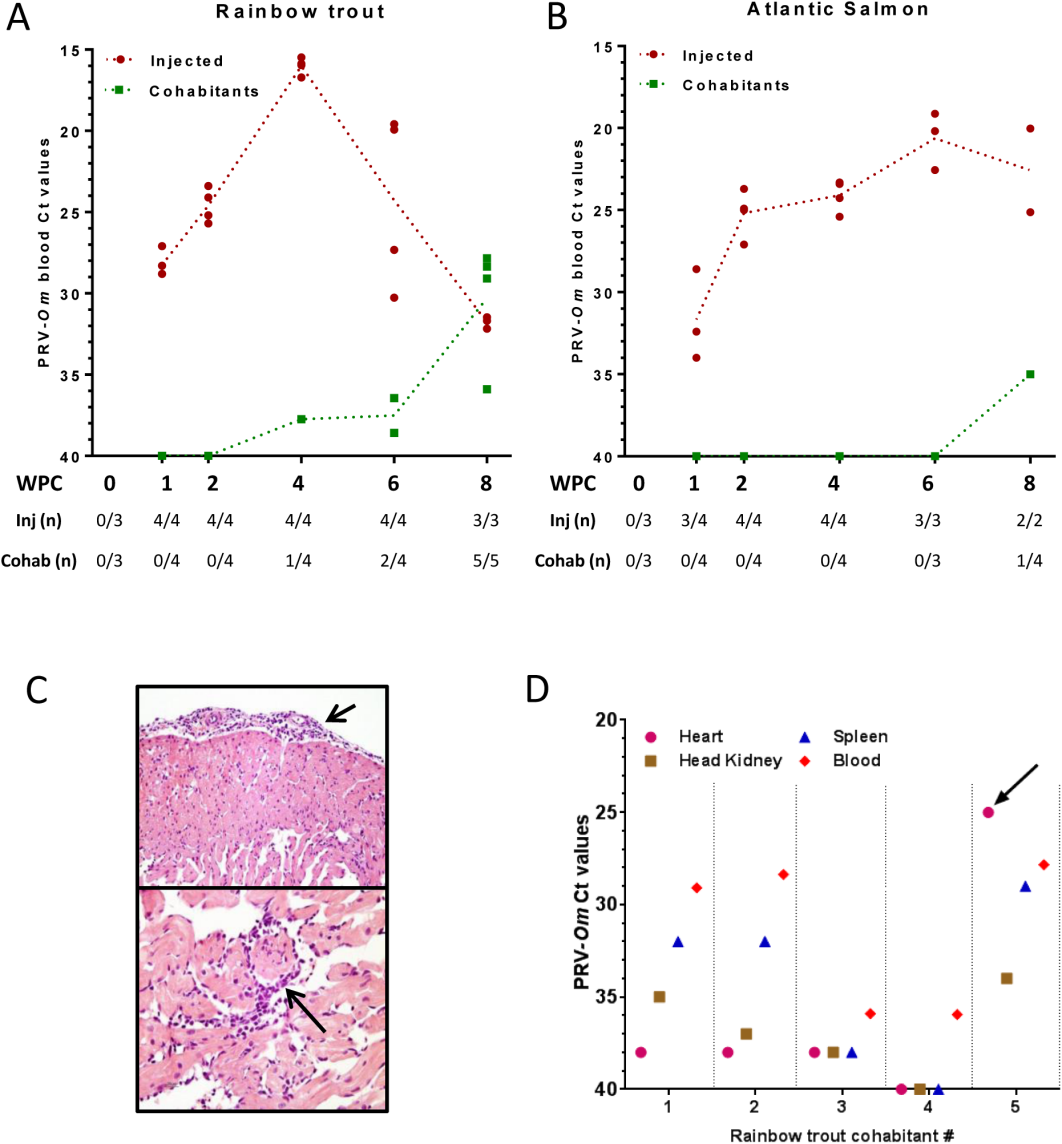
Rainbow trout and Atlantic salmon short term study (Trial 1): PRV-*Om* in blood, histopathology and tissue distribution. Virus analysis was performed by RT-qPCR targeting the PRV-*Om* segment S1 on blood from A) rainbow trout and B) Atlantic salmon. Large dots indicate Ct value of individual fish and dotted trend lines the mean Ct value of virus-positive fish. Virus-injected fish (red) and cohabitants (green) are shown. The table below the graphs shows the number of virus positive fish and examined fish per sampling point. C) Epicarditis (short arrow) and endo- and myocarditis (long arrow) in rainbow trout cohabitant # 5, 8 WPC. D) Ct values of PRV-*Om* RNA in organs from five individual rainbow trout cohabitants 8 WPC. The heart value of the individual with heart pathology (# 5) is marked by arrow.

All injected rainbow trout tested positive for PRV-*Om* RNA in blood by RT-qPCR at all samplings from 1 to 8 WPC. The virus levels peaked at 4 WPC (mean Ct value 16.0) demonstrating that an effective replication of PRV-*Om* occurred following injection (Fig 1A, S1 Data file). The virus RNA levels then declined in blood, reaching a mean PRV-*Om* Ct value of 30 at 8 WPC. All rainbow trout cohabitants were confirmed virus positive by RT-qPCR at 8 WPC, demonstrating a horizontal transmission of PRV-*Om* between rainbow trout.

Similarly, all Atlantic salmon injected with PRV-*Om* challenge material were also virus positive in blood by RT-qPCR throughout the trial period (Fig 1B, S1 Data file). However, the PRV-*Om* RNA levels increased at a slower rate, with a less defined peak phase (mean Ct 20.6 at 6 WPC) and no obvious decrease at 8 WPC. The transmission of virus to cohabitants could not be concluded on for Atlantic salmon, as only one out of four cohabitants sampled at 8 WPC tested positive for PRV-*Om* in blood (Ct 35). In this experiment, no clinical disease was observed, and no mortality occurred in any of the salmonid species.

Histological examination of rainbow trout revealed mild heart inflammation in one injected fish sampled at 6 WPC and one cohabitant fish sampled at 8 WPC. In the latter fish, epicarditis and focal endo- and myocarditis in atrium and stratum spongiosum of the heart ventricle was confirmed (Fig 1C). No lesions were seen in the additional tissues examined. No pathological findings were detected by the histological examination of Atlantic salmon in this experiment.

To explore tissue tropism of the virus, rainbow trout cohabitants sampled at 8 WPC were analysed for PRV-*Om* RNA levels by RT-qPCR in the heart, spleen and head kidney (Fig 1D). The highest levels were obtained in blood for four out of five fish, followed by spleen, head kidney, and finally heart. Interestingly, the individual confirmed to have pathological changes in the heart also turned out to have higher PRV-*Om* RNA levels in the heart (Ct 25) compared to blood. For virus-injected fish the data show that in the viraemic phase at 4 WPC blood exhibited higher PRV-*Om* RNA levels compared to other organs, whereas later in the infection cycle at 8 WPC, the virus appeared to accumulate in heart (S1 Fig).

Based on previous reports showing that PRV*-Ss* replicates in the circulating red blood cells of Atlantic salmon [10], and the finding of high PRV-*Om* RNA levels in blood of diseased rainbow trout from the field as well as the experimental trial, blood smears from the rainbow trout field samples used as challenge material (Hatchery D, [2]) were analysed by immunofluorescence staining. PRV-*Ss* σ1 and μ1c antiserum did not show positive staining on this material, but when using a double-stranded RNA-binding antibody, intracellular inclusion bodies resembling those previously reported for PRV-*Ss* were observed (S2 Fig).

### Trial 2, rainbow trout long-term study

**Fig 2:**
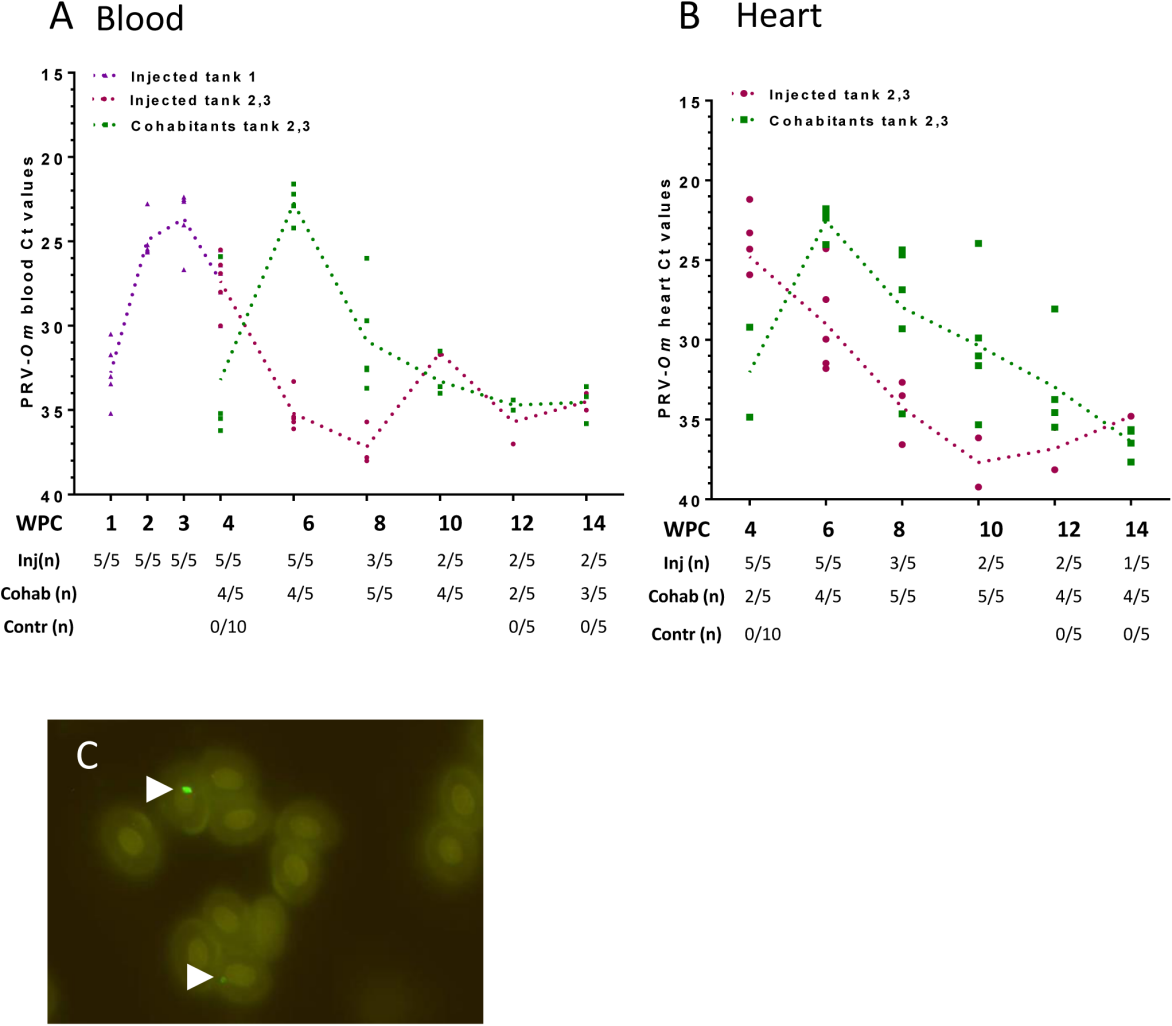
Rainbow trout long-term study (trial 2): PRV-*Om* in blood and heart. A) Virus analysis performed by RT-qPCR targeting PRV-*Om* segment S1 in blood from virus-injected fish from three tanks (Tank 1: purple, parallel tanks 2 and 3: red) and cohabitants from tanks 2 and 3 (green). Large dots indicate Ct values of individual fish and dotted trend lines the mean Ct value of virus-positive fish. The table below shows number of positive fish per sampling point. B) PRV-*Om* Ct values in heart of fish from tanks 2 and 3. C) Detection of PRV-*Om* in rainbow trout red blood cells using rabbit antiserum targeting PRV-*Ss* σ1. Arrows mark positive staining.

To explore infection dynamics, pathology and development of disease in rainbow trout, a 14 weeks study was performed (trial 2, Table 1). The infection developed with similar kinetics in the PRV-*Om* injected fish and in the cohabitants as in the former 8 weeks trial, and was characterized by an acute phase with a defined viral peak in blood and a subsequent drop (Fig 2A, S2 Data file). In the injected fish, the viral loads as measured by RT-qPCR reached the highest level in blood 3–4 WPC (mean Ct 23.7, 5 out of 5 positive fish). Then the number of fish with detectable virus levels decreased with increasing Ct values throughout the course of the experiment. In cohabitants, the infection in blood peaked 6 WPC (mean Ct 22.7, 4 out of 5 fish positive), after which the viral RNA load decreased over the course of the trial period, as observed for the injected fish. PRV-*Om* levels in heart tissue increased in concert with blood levels (Fig 2B), and the lowest Ct levels were measured in injected fish at 4 WPC (mean 24.7, 5 out of 5 fish positive) and at 6 WPC in cohabitants (mean 22.5, 4 out of 5 fish positive). The virus transmission efficiency to cohabitants was 100% at 8 WPC, and 80% at 4, 6 and 10 WPC.

During this experiment, one virus-injected fish died 25 days post challenge and the infection with PRV-*Om* was confirmed by RT-qPCR on heart (Ct 26.8), spleen (Ct 24.9) and kidney tissues (Ct 23.6). Gross pathological findings included pale gills, ascites and haemorrhages (S3 Fig), consistent with those described for field outbreaks [2]. Bacteriological analysis gave negative results.

Staining of infected blood cells from five cohabitants from 6 WPC with antiserum targeting the PRV-*Ss* σ1 protein (Fig 2C) revealed cytoplasmic inclusions resembling the viral factories previously described for PRV-*Ss* in Atlantic salmon erythrocytes [10,16], in four out of five individuals. The negative cohabitant was negative for PRV*-Om* RNA. Control samples negative for PRV RNA did not show any background staining. The inclusions were also similar to those observed by staining of dsRNA in rainbow trout from a field outbreak (S2 Fig).

**Fig 3:**
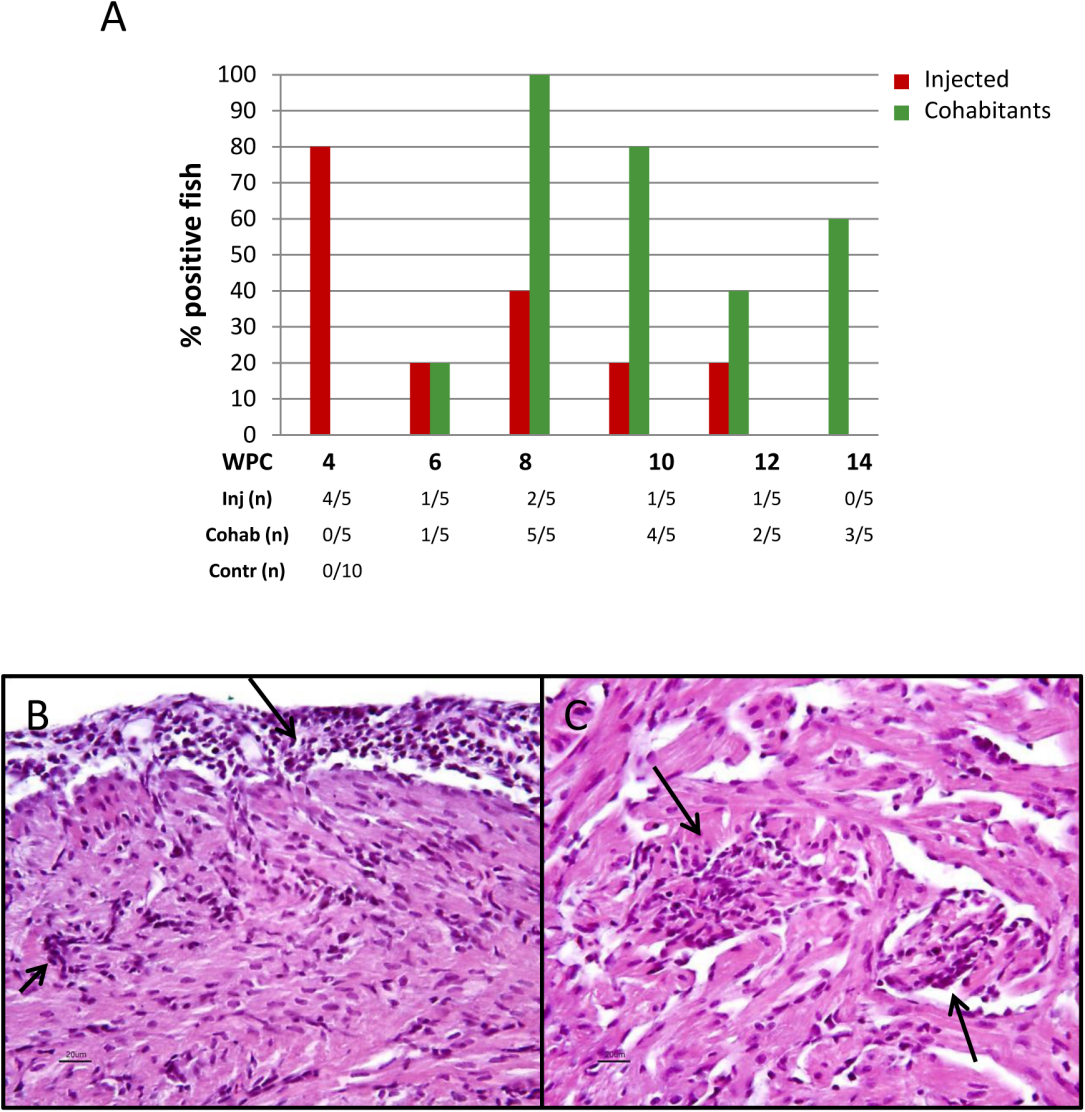
Rainbow trout long-term study (trial 2): Histopathological findings. A) Prevalence of rainbow trout with heart pathology after injection of PRV-*Om* (red) and in cohabitants (green). Five fish were sampled from each group at each time point. The table below shows the number of positive fish out of total number examined. B–C) Light microscopic images of heart of a rainbow trout cohabitant (8 WPC). B) Epicarditis (long arrow) and perivasculitis in the compact layer of the ventricle (short arrow). C) Focal endo- and myocarditis in the spongious layer of the ventricle (arrows).

Histopathological findings in heart were observed in 24 out of the 60 exposed rainbow trout examined. The prevalence and distribution of heart lesions among i.p. injected fish and cohabitants at each time point is shown in Fig 3A. In general, the prevalence of lesions in the heart was higher among cohabitants compared to injected fish. The lesions observed in the heart were mild to moderate and comprised epicarditis, focal endo- and myocarditis in *stratum spongiosum* of the ventricle and sometimes also in the atrium. In some hearts perivasculitis and myocarditis in *stratum compactum* of the ventricle were also observed (Fig 3B and 3C). The most prominent lesions were seen in cohabitants at 8 WPC, where one fish had mild lesions, three fish moderate, and one fish had severe heart pathology. Otherwise most of the positive hearts (17 out of 19) were characterized as mildly affected. One cohabitant at 10 WPC had a few degenerated red muscle fibers in addition to heart pathology. Otherwise, no findings were observed in the muscle and liver samples examined, and immunohistochemical examinations gave negative results. Histological examination of heart from non-exposed fish showed no lesions as described in exposed fish. The disease incidence rate was 100% at 6 WPC and 80% at 8 WPC, which is in line with the infection rate at these two time points.

A crowding stress test was performed in one tank of infected rainbow trout at 10 WPC, but at the time the infection had passed peak viraemia, and only 50% of the fish were still virus positive. No differences were observed compared to the other infected groups.

### Trial 3, Atlantic salmon long-term study

**Fig 4:**
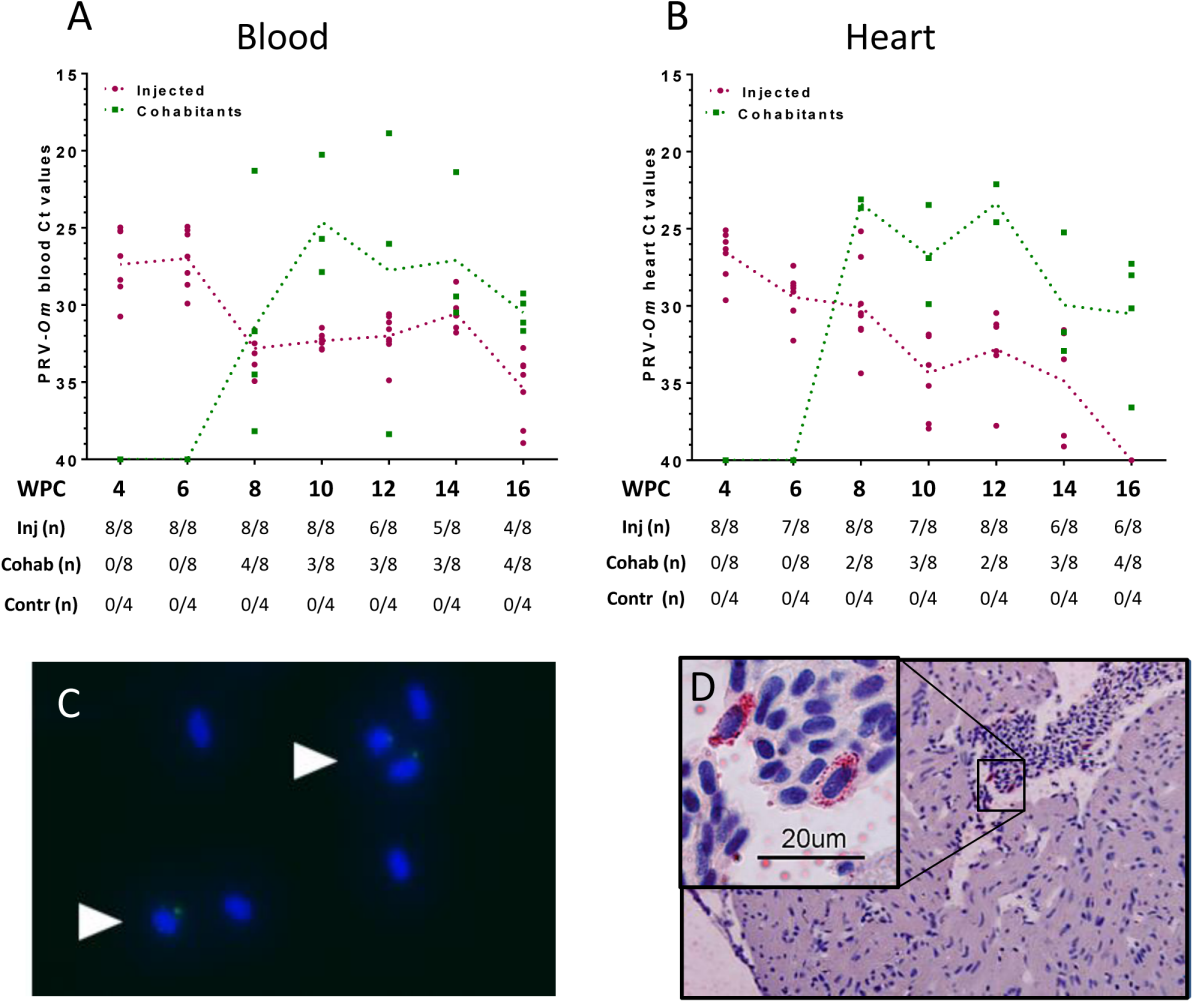
Atlantic salmon long-term study (trial 3): PRV-*Om* in blood and heart. A) Virus analysis performed by RT-qPCR targeting segment S1 in blood from virus-injected fish (red) and cohabitant fish (green). Large dots indicate the Ct value of individual fish and dotted trend lines the mean Ct value of virus-positive fish. The table below shows the number of positive fish per sampling point. B) PRV-*Om* Ct values in heart. C) Detection of PRV-*Om* in erythrocytes using rabbit antiserum targeting PRV σ1. Arrows point to cells with PRV-*Om* inclusions (green). The cell nuclei are dyed blue (Hoechst). D) Detection of dsRNA inclusions in erythrocytes in lumen of the heart ventricle using an antibody targeting dsRNA.

In the Atlantic salmon long-term study (trial 3, Table 1), the virus-injected shedder fish did not display a defined virus peak in blood as observed in rainbow trout. The highest levels of virus were detected 4 and 6 WPC (mean Ct 27.3 and 27.2, respectively), declining towards virus Ct levels around 30 from 8 WPC and onwards (Fig 4A, S3 Data file). In cohabitants, PRV-*Om* was first detected in four out of eight fish at 8 WPC and at all subsequent sampling points three or four out of eight fish were virus positive. Four individual cohabitants sampled at different time points reached Ct levels below 22 (Ct 18.9–21.4) in blood, indicating that even if the number of infected Atlantic salmon was low compared to rainbow trout, the virus replicated well in blood. In heart, the peak viral load was observed at 4 WPC in injected fish (mean Ct 26.5), and then slowly declined until undetectable levels at 16 WPC (Fig 4B). Most infected cohabitant fish were also virus positive in heart, and between two and four out of eight fish were positive at each sampling point throughout the study. With regards to heart samples, five fish sampled at different time points reached virus levels below Ct 25, of which four also had high levels of PRV-*Om* in blood. For Atlantic salmon the virus transmission rate to cohabitants was 40–50% between 8 and 16 WPC.

Similar to findings in infected rainbow trout, intracellular inclusions containing viral proteins were observed in Atlantic salmon erythrocytes (Fig 4C), indicating that PRV-*Om* infects Atlantic salmon and rainbow trout erythrocytes in the same manner as PRV-*Ss*. Immunohistochemical examinations in hearts using PRV antibodies were negative. However, when using antibodies against dsRNA, positive staining was detected in the cytoplasm of single erythrocytes in lumen of the heart ventricle in one fish (Fig 4D). No staining was observed in myocytes.

**Fig 5:**
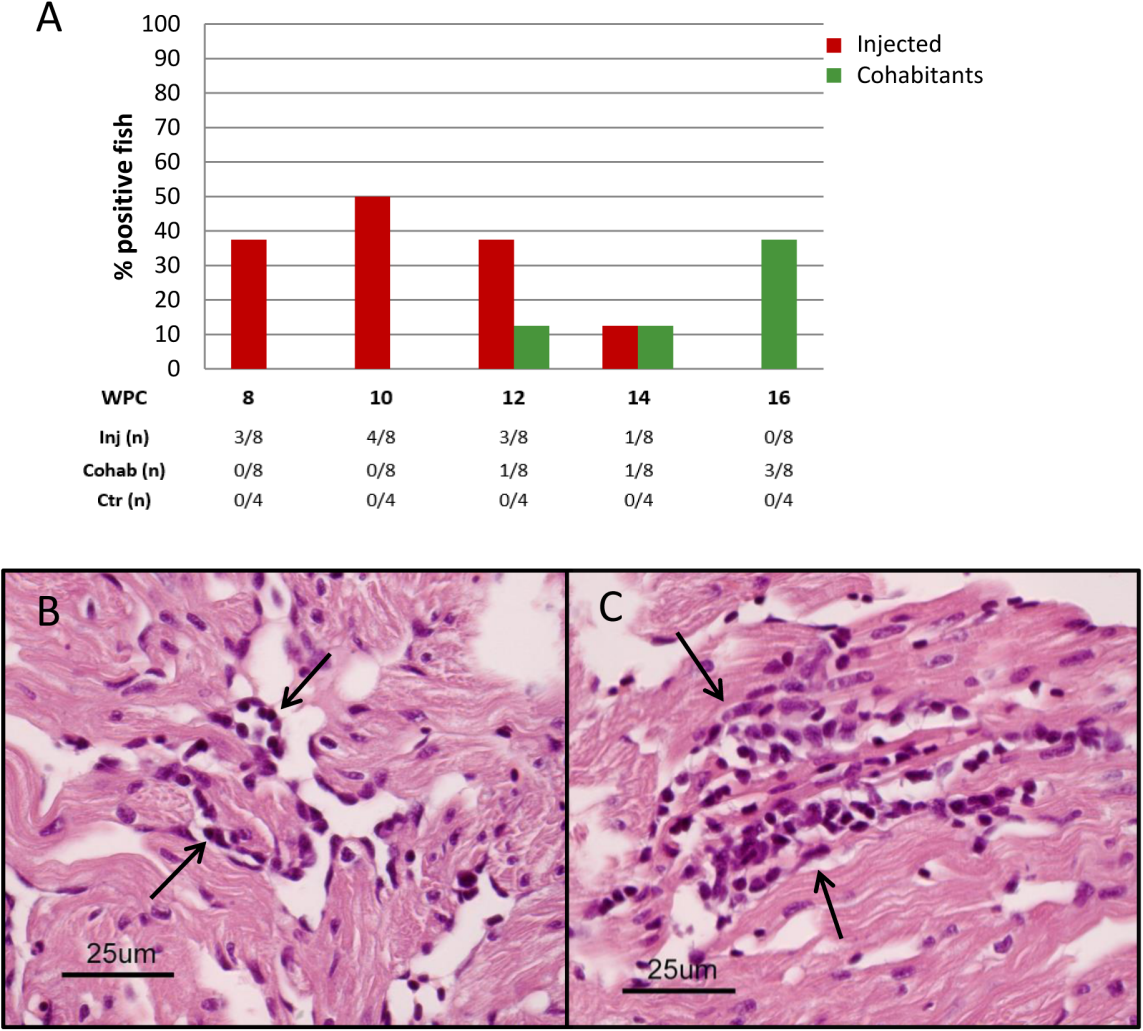
Atlantic salmon long-term study (trial 3): Histopathological findings. A) Prevalence of Atlantic salmon with focal myocarditis in heart ventricle of PRV-*Om* injected and cohabitant fish. Eight fish from each group were examined at each sampling. The table below shows the number of positive fish out of total number examined. B-C) Light microscopic images of an Atlantic salmon heart ventricle 12 weeks after injection of the virus. B) Mild focal endo- and myocarditis (arrows). C) Mild but more distinct focal myocarditis (arrows).

No clinical disease was observed in infected Atlantic salmon, and no mortality occurred during the study. However, mild focal myocarditis was detected in less than 50% of the virus-injected individuals between 8 and 14 WPC, with the highest number of affected individuals at 10 WPC. For cohabitants, similar changes were detected at an incidence rate of 10 to 40% between 12 and 16 WPC (Fig 5).

The Atlantic salmon cohabitants exposed to crowding and hypoxic stress did not display significant differences from the non-stressed cohabitants (S3 Data file), but some trends were observed (S4–S6 Figs). In particular, the prevalence of infected fish was higher in the stressed cohabitants at 16 WPC (S5 Fig). The stress did not cause more severe lesions or increase the number of individuals with pathological changes.

No anaemia or decreased levels of haemoglobin were observed in any of the infected salmon groups (S6A Fig). Haematocrit and haemoglobin measures correlated well in this trial (S6B Fig).

### Innate antiviral responses in rainbow trout and Atlantic salmon

**Fig 6:**
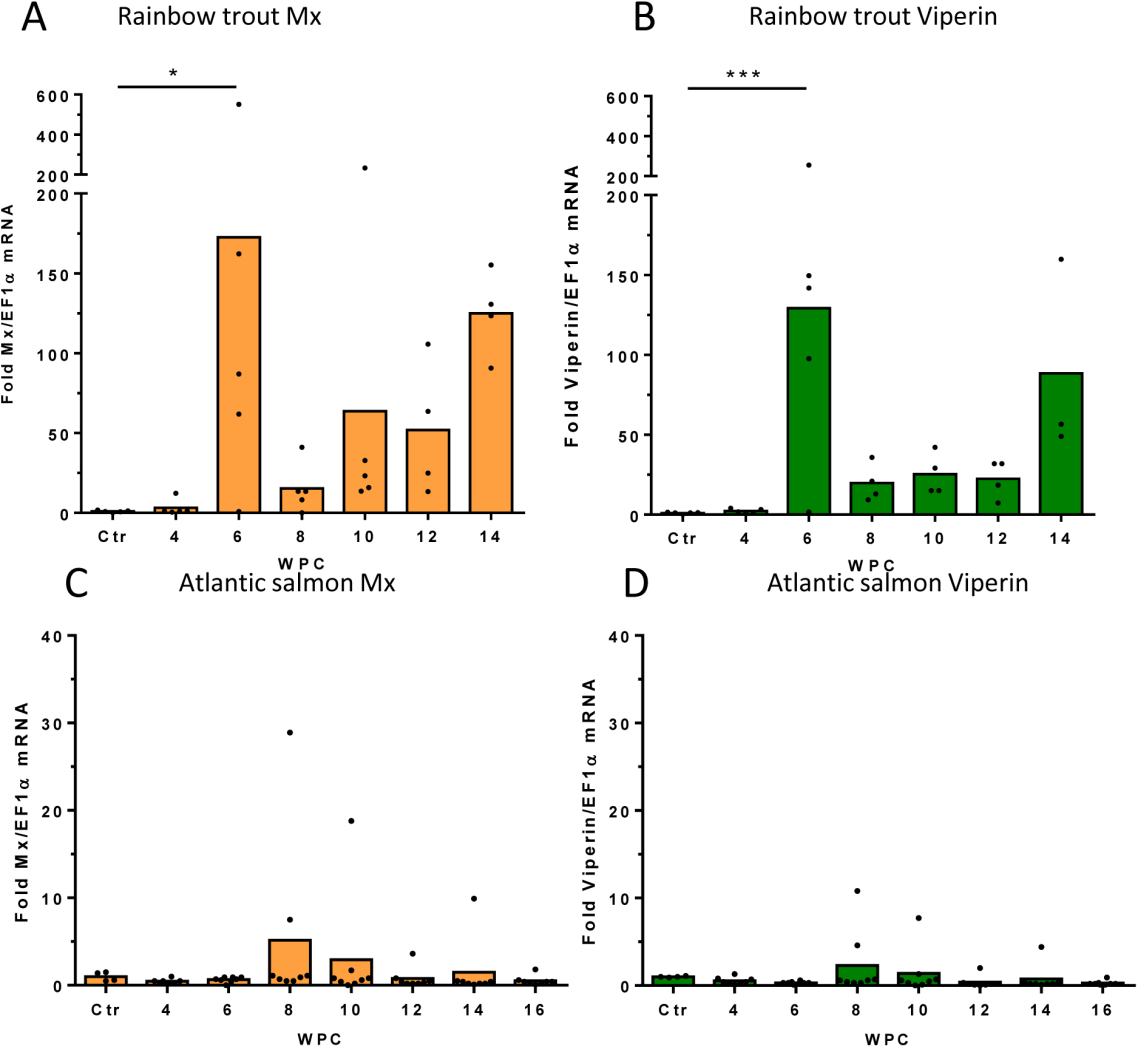
Spleen antiviral responses to PRV-*Om*. Fold increase in expression of the interferon-regulated antiviral genes Mx and viperin during the course of infection with PRV-*Om* in rainbow trout cohabitants (A, B) and Atlantic salmon cohabitants (C, D) from the two long-term trials (trial 2 and 3). Individual values of fold induction and bar at mean is shown. Significant differences from control fish are shown * P<0,05, *** P<0,001.

To address the innate antiviral responses to the infection in both species, spleen samples from cohabitants from the two long-term trials (trials 2 and 3, S4 Data file) were analysed for expression of antiviral genes Mx and viperin. Rainbow trout responded significantly to the viral infection at 6 WPC, and the maximum antiviral gene levels corresponded to the virus peaks in blood and heart (Fig 6A and 6B). A mean 40-fold increase in Mx expression and a mean 130-fold increase in viperin expression, was observed relative to levels in uninfected fish. The expression then decreased along with virus levels. A second increase in Mx and Viperin levels were observed at 14 WPC, but this second boost was not associated with increased levels of PRV-*Om*.

Compared to rainbow trout, Atlantic salmon responded very weakly to PRV-*Om* infection. Four individuals, all positive for PRV-*Om*, showed 5–30 fold induction of Mx and only two individuals showed 5–10 fold induction of viperin. The remaining cohabitants showed no increase in Mx or viperin expression.

## Discussion

Due to the emergence of this novel PRV variant in Norway, it was of primary importance to assess the risk associated with PRV-*Om* infection in rainbow trout and Atlantic salmon. To do so, it was necessary to establish a challenge model under controlled conditions, to evaluate the transmissibility of the virus from infected fish to naïve cohabitants, to assess the pathogenicity of the novel variant in farmed salmonids and provide scientific based evidence for improved diagnostics.

The results from these experimental trials strongly suggest that PRV*-Om* causes heart pathology in rainbow trout, and strengthens the indications that PRV*-Om* was the primary cause of the disease outbreaks reported in Norwegian rainbow trout hatcheries in 2013, when the virus was first detected [2].

In this study, two separate experiments (trial 1A and 2) consistently show that PRV-*Om* efficiently replicate in rainbow trout blood after i.p.-injection, reaching an acute peak phase after 3–4 weeks. PRV*-Om* levels in the rainbow trout hearts corresponded well with blood levels. The naïve cohabitant fish were infected directly after the virus peak in the injected fish, demonstrating rapid horizontal transmission of the virus and suggesting that the virus is shed during the peak phase. It has previously been shown by experimental challenge that PRV-*Ss* can infect Atlantic salmon through the intestinal wall and shed to faeces [21]. This could be an infection- and transmission route also for PRV-*Om*, but this hypothesis needs further investigation.

The heart inflammation observed in the rainbow trout was considered mild to moderate compared to the field cases described previously [2], but with similar characteristics. The prevalence of lesions in the heart was higher among rainbow trout cohabitants compared to virus-injected fish in the trial. This finding may indicate that the natural horizontal infection route may trigger stronger inflammation. Previous studies in Atlantic salmon suggest that CD8-positive lymphocytes constitute the majority of the cellular infiltrates in HSMI affected hearts [22,23], and this may represent a cytotoxic attack on virus-infected myocytes. In line with this, infected myocytes are clearly reduced in number after the onset of inflammation, as previously demonstrated by immunohistochemical staining [24]. Expression of cytotoxic effector genes like granzyme has also been reported in HSMI hearts [22,23]. Similar mechanisms of disease onset may apply for PRV*-Om*, and should be subject to further study.

Two virus-injected rainbow trout in trial 2 were found dead 3 WPC,. In both fish, viral RNA was detected in heart and blood, but no histological analysis was performed. The time point (3 WPC) corresponds to the peak viraemia phase in the long-term trial. Taken together, the results strengthen the proposed association between PRV-*Om* and disease in rainbow trout.

The two experiments in Atlantic salmon (trial 1B and 3) both demonstrated that PRV-*Om* also replicates in this species, although the infection dynamics differed from rainbow trout. Whereas virus RNA levels peaked sharply in virus-injected rainbow trout, a plateau level was reached at 4–6 WPC in Atlantic salmon, indicating a slower replication rate. When comparing the infection kinetics in trial 2 and 3 one should be aware that there were small differences in the preparation of challenge material and blood analyses of the two studies. However, the same characteristic differences between the injected groups were seen when comparing trial 1A and 1B, which were performed simultaneously with identical procedures. The Atlantic salmon long-term trial demonstrated that Atlantic salmon cohabitants could also be infected, confirming susceptibility to the virus through a horizontal infection route also for salmon. However, the transmission was less effective and only about 50% of the cohabitants were infected between 8 and 16 WPC, indicating that PRV-*Om* is less adapted to Atlantic salmon. The lower transmission rate could be due to several mechanisms. One possibility is less effective virus shedding. It has previously been reported that blood levels of PRV-*Ss* reflects the release of virus into faeces [21], and PRV-*Om* do not reach as high peak levels in Atlantic salmon blood as in rainbow trout. More likely, however, the virus entry into salmon cohabitants is less effective due to differences in virus-receptor interactions between the two species. So far, the receptor for PRV is not known. Mammalian orthoreovirus (MRV) use junction adhesion molecule (JAM)–A as its main receptor for virus entry [25,26]. However, the amino acid sequences reported to interact with receptors are not conserved between MRV and PRV, and the receptor and entry mechanisms may be very different for PRV [6,27]. MRV is also highly dependent on the enzymatic conditions in the intestinal tract for optimal infection efficiency [28], and the intestinal environment may also differ between the species and be central for transmission for PRV-*Om*.

The lack of a defined peak phase and slower rate of virus clearance in Atlantic salmon blood and heart may indicate that Atlantic salmon handles the PRV-*Om* infection differently from rainbow trout also after the initial infection. The antiviral response data obtained in the two long-term trials suggests an explanation. The Atlantic salmon responded poorly to PRV-*Om* infection with regards to antiviral gene expression (Mx and viperin) compared to rainbow trout. In contrast, PRV-*Ss* generally induces Mx and viperin responses in Atlantic salmon [29,30], but this does not seem to eliminate the PRV infection. Induction of interferon-regulated genes like Mx and viperin is commonly the first step in limiting viral replication and mounting a more specific immune response which may clear the virus [31].

Atlantic salmon in the current study developed considerably milder histopathological changes in response to PRV-*Om*, as compared to rainbow trout. In contrast, Norwegian pathogenic isolates of PRV-*Ss* induce a strong and long-lasting antiviral response [29,30]. A North American PRV-*Ss* isolate, however, has not been associated with HSMI pathology in Atlantic salmon or Sockeye salmon (*Onchorhynchus nerka*) infection experiments [32]. Recently, a new infection study in Sockeye salmon using the North American PRV isolate showed that the early antiviral response to infection was very limited [33]. This may indicate that the early antiviral response plays an important role in triggering pathology, and the very low antiviral response to PRV-*Om* in Atlantic salmon could be the reason why the pathological findings were minor. It should be noted that our results would have been strengthened if the fish had been separated into parallel tanks to avoid tank-specific effects on the immune response.

Field observations indicate that mortality due to HSMI in Atlantic salmon are linked to situations where the fish were exposed to additional stress like transport, crowding, pumping and delousing. Based on data on virus kinetics from the first experiment, a stress test was performed at 10 WPC in the Atlantic salmon long-term trial when infection was predicted to be in an early phase in the cohabitants. There was a trend towards a higher number of infected fish in the stressed group, but mean levels of infection did not differ significantly, and no induction of pathology or anaemia was observed in the stressed salmon.

When comparing PRV-*Om* infection in rainbow trout with studies on PRV-*Ss* infection in Atlantic salmon, it is interesting to note that the organs and cells targeted by the virus are similar (heart tissue, red blood cells). As previously shown for PRV*-Ss* in Atlantic salmon [10,16], PRV-*Om* infects erythrocytes, as demonstrated by immune staining using antiserum directed against PRV*-Ss* σ1 [9]. This outer capsid protein is responsible for binding of reovirus to target receptors [6,34,35]. The staining pattern revealed distinct globular cytoplasmic inclusions in erythrocytes from both rainbow trout and Atlantic salmon. The inclusions probably represent viral factories, as previously reported for PRV*-Ss* and MRV [10,16,36,37]. It should be noted that both the experimental fish and the challenge material tested negative for PRV-*Ss* by RT-qPCR, and that the positive PRV-*Om* staining in erythrocytes correlated with low PRV-*Om* Ct levels in the same blood sample, making any false positive staining unlikely. This finding indicate that erythrocytes are important for PRV*-Om* replication, which may also serve as an explanation for the haemolytic anaemia described in field outbreaks [2]. Anaemia was not found in our trials, and is not commonly observed in HSMI outbreaks. However, a PRV variant found in Coho salmon in Japan was recently associated with Erythrocytic Inclusion Body Syndrome (EIBS) [38], a disease characterized by anaemia. In light of this, it is not unlikely that the anaemia observed in the field is mediated by PRV-*Om*, even if we did not observe anaemia in our experimental studies.

PRV-*Ss* can be cultured in primary erythrocytes *ex vivo* [16], and although virus is released from infected cells to the culture media, no erythrocyte loss or lysis have been observed in culture. Induced expression of erythropoietic genes has been observed in head kidney during PRV*-Ss* infection [29], indicating an increased formation of new blood cells during the course of disease. These findings could explain why anaemia is not a common finding in PRV-infected Atlantic salmon, and why we did not detect anaemia in our PRV-*Om* trials. The anaemia observed in hatchery outbreaks may be due to a secondary factor triggering a more severe disease [2]. Even if the PRV*-Ss* antiserum was able to cross-react with PRV*-Om* in infected blood from both Atlantic salmon and Rainbow collected at the peak phase, the sensitivity appeared to be low as staining was only detectable in blood samples with PRV-*Om* Ct levels below 20. The inclusions were smaller and less numerous compared to the multiple large inclusions previously observed in the peak phase of PRV-*Ss* infection in Atlantic salmon [16]. Furthermore, staining with a dsRNA antibody in a blood smear from a PRV*-Om* outbreak and in a heart sample from the Atlantic salmon trial 3, led to detection of several cytoplasmic inclusions per erythrocyte. Since dsRNA detection is not specific for PRV, it is uncertain if these inclusions all contained PRV-*Om* RNA. Unfortunately, it was not possible to establish a link between observed heart lesions and PRV infection of cardiomyocytes using the available PRV-*Ss* antibodies [9], probably due to a lower affinity of these antibodies for PRV*-Om*.

Both PRV-*Ss* in Atlantic salmon and PRV-*Om* in rainbow trout elicit a strong antiviral interferon mediated immune response. This is documented in PRV-*Ss* in Atlantic salmon experimental challenge where the antiviral responses has been characterized as long-lasting [30], and able to protect the fish from secondary infection by other viruses for months [39].

Interestingly, PRV-*Ss* in Atlantic salmon tends to establish a persistent infection [10]. In the infection trial performed with the North American PRV-*Ss* variant in Atlantic salmon, virus RNA-levels persisted in blood for up to 59 weeks [32]. and also in the field situation, farmed Atlantic salmon tend to stay PRV positive. On the contrary, under experimental infection PRV-*Om* behaves like an acute infection in rainbow trout characterized by a viral peak and subsequent clearance. Further studies need to be performed to explain this different host-pathogen interaction.

Although Atlantic salmon is less susceptible to PRV-*Om*, our findings implicate that since since it is possible to detect PRV-Om RNA in infected Atlantic 14 WPC, they could serve as a natural reservoir for PRV-*Om* in locations hosting both salmon and rainbow trout. This should be further investigated. An interesting question is also if PRV-*Ss* and PRV-*Om* co-infection can lead to exchange of viral genomic segments (virus reassortment), creating new PRV-variants. Reassortment has previously been demonstrated to lead to evolutionary changes in other segmented RNA viruses, like the Bluetongue virus [40], and is also described for betanodaviruses in fish [41].

Preliminary analysis of tissue tropism in the 8 weeks study in rainbow trout indicated that PRV-*Om* reaches the highest levels in blood and spleen in the early phase of infection, but could potentially accumulate in the heart in the later phase. Similar observations have been made for PRV-*Ss* [10], although PRV-*Om* levels appear to be more effectively reduced in rainbow trout blood compared to PRV-*Ss* in Atlantic salmon blood. Also, high levels of PRV-*Om* in heart were more closely associated with the onset of heart pathology. Results from trial 1 were consistent with the long-term trials, suggesting that heart and spleen, or blood itself, are all suited for diagnostic testing for PRV-*Om*. and high virus levels in these tissues coincides with pathology and possibly mortality. This should be evaluated more closely in future experimental studies and on field cases. The development of sensitive antibodies or in situ hybridization assays specific for PRV-*Om* will also be helpful as diagnostic tools to study the localization of the virus in target organs.

So far, outbreaks associated with PRV-*Om* has only been reported in farmed rainbow trout in Norway 2013–2014 in fish with a common origin [2]. A screening program was initiated in 2016 to map the incidences in the area where infected fish were set to sea (http://www.vetinst.no/rapporter-og-publikasjoner/rapporter/2016/fish-health-report-2015). Due to the relatively low virulence of this pathogen, its presence and impact might be underestimated. Therefore it is highly recommended that the putative presence of PRV-*Om* in other countries farming rainbow trout is further investigated. Recently, three other findings of PRV-variants have been reported. Firstly, a sequence resembling PRV-*Om* was detected in coho salmon (*Oncorhynchus kisutch*) in Chile [42], another PRV variant was associated with Erythrocyte Inclusion Body Syndrome (EIBS) in Coho salmon in Japan [38], and a more distantly related orthoreovirus was found associated with disease in largemouth bass (*Micropterus salmoides*) [43]. This indicates that orthoreoviruses in fish may be more prevalent, and much more diverse, than originally anticipated.

PRV viruses are challenging to produce and purify *ex vivo*, since there are not any available cell lines that support effective PRV-infection and replication. Hence, we have not been able to confirm that PRV-*Om* is the sole causative agent for the disease in rainbow trout. Nevertheless, several lines of evidence from the challenge trials strongly suggest that PRV-*Om* is the causative pathogen; 1) An increase of viral RNA in fish injected with infected blood cells, 2) transmission of virus and establishment of infection in naïve cohabitants, 3) staining of cytoplasmic structures in erythrocytes, and finally 3) the association between infection and the development of histopathological lesions in target organs, which were similar to findings in field outbreaks.

Based on these challenge trials, it can be concluded that PRV-*Om* replicates in rainbow trout erythrocytes, transmits horizontally, and is associated with heart pathology. PRV*-Om* also infects and transmits horizontally between Atlantic salmon, although with a slower replication kinetics and transmission, and the pathological consequences was minor under experimental conditions.

## Supporting information

S1 Fig Trial 1A-Tissue distribution of PRV*-Om* RNA. Virus analysis was performed by RT-qPCR targeting the PRV-*Om* segment S1 on heart, head kidney, spleen and blood) from virus-injected rainbow trout sampled at 0, 4 and 8 WPC. Indvidual Ct values (colored dots) and mean Ct levels (horizontal line) are shown.
(TIF)

S2 Fig Detection of dsRNA in erythrocytes from PRV-*Om* infected rainbow trout. Staining of double stranded RNA was performed on blood smears from rainbow trout sampled in a field outbreak from 2014. Blood smears were air dried before fixation in ice cold methanol. Slides were then rehydrated in PBS and blocked in PBS with 5% skimmed milk before staining with monoclonal antibody against dsRNA (J2; 1:200) (Scicons, Hungary), washed and stained with secondary antibody; goat anti-mouse Alexa Fluor 595 (1:1000) from Molecular Probes (Life Technologies). The nuclei were stained with Hoechst 33342 (Life technologies). Coverslips were mounted with Fluoroshield (Sigma-Aldrich) and visualized on an inverted fluorescence microscope (Olympus IX81).
(TIF)

S3 Fig Trial 2 -Gross pathology. Injected rainbow trout 25 days post challenge. A) abdominal distension and pale gills. B) ascites and anaemia of internal organs. C) focal haemorrhage on abdominal wall.
(TIF)

S4 Fig Trial 3- weight curve. Weight curves based on the eight fish sampled per group at each time-point. Uninfected controls (grey), virus-injected fish (red), cohabitants (green) and stressed cohabitants (blue) are shown. Large dots represent individual measurements and the dotted trend lines show the group mean. The stressed cohabitants were exposed to an oxygen saturation of 40–50% for 4 h at 10 WPC and sampled at 12, 14 and 16 WPC.
(TIF)

S5 Fig Trial 3-Effects of hypoxic stress on virus RNA detection in blood and heart in Atlantic salmon. Virus analysis were performed by RT-qPCR targeting the PRV-*Om* segment S1 on blood (A) and heart (B) from cohabitants sampled at 10 WPC and cohabitants (open bars) and stressed cohabitants (blue bars) at 16 WPC/end of trial. The stressed cohabitants were exposed to an oxygen saturation of 40–50% for 4 h at 10 WPC. Dots represent individual Ct-values and the line show the group mean. The number of positive fish and total fish sampled are given below the figure (Cohab (n)).
(TIF)

S6 Fig Trial 3 –haemoglobin/haematocrit curve and correlation. A) hemoglobin levels based on the eight fish sampled per group at each time-point. Uninfected controls (grey), virus-injected fish (red), cohabitants (green) and stressed cohabitants (blue) are shown. Large dots represent individual measurements and the dotted trend lines show the group mean. The stressed cohabitants were exposed to an oxygen saturation of 40–50% for 4 h at 10 WPC. The stress tested fish were sampled at 12, 14 and 16 WPC. Hemoglobin levels were measured using a HemoCue Hb 201+ with corresponding cuvettes, performed according to the manufacturers protocol (HemoCue). B) Correlation between hematocrit and hemoglobin levels measured in all groups. Hematocrit was measured using the standard procedure with micro haematocrit tubes (GMBH) and haematocrit centrifugation directly after blood sampling.
(TIF)

S1 Table Statistical power test of trial 2 and 3. (DOCX)

S1 Data file Trial 1. (XLSX)

S2 Data file Trial 2. (XLSX)

S3 Data file Trial 3. (XLSX)

S4 Data file Antiviral immune response qPCR data. (XLSX)
