# Supplementary figures and images for: Infection experiments with novel Piscine orthoreovirus from rainbow trout (Oncorhynchus mykiss) in salmonids
Source: PLoS One. 2017 Jul 5;12(7):e0180293. doi: 10.1371/journal.pone.0180293 (PMC5497981; doi:10.1371/journal.pone.0180293)

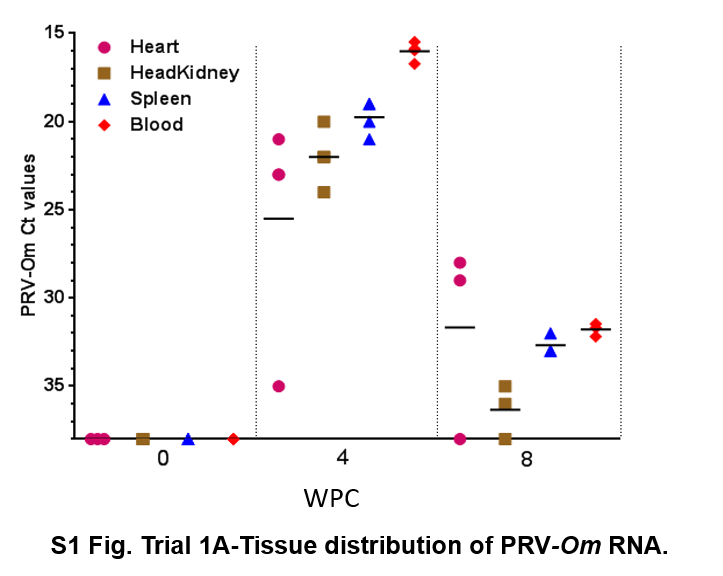

Supplement: S1 Fig — Virus analysis was performed by RT-qPCR targeting the PRV-Om segment S1 on heart, head kidney, spleen and blood) from virus-injected rainbow trout sampled at 0, 4 and 8 WPC. Indvidual Ct values (colored dots) and mean Ct levels (horizontal line) are shown. (TIF) [file pone.0180293.s001.tif]

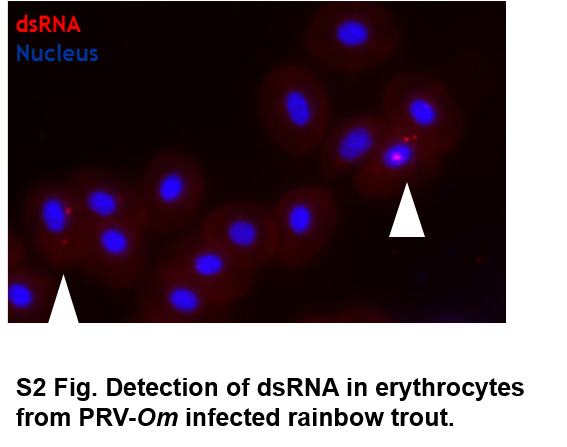

Supplement: S2 Fig — Staining of double stranded RNA was performed on blood smears from rainbow trout sampled in a field outbreak from 2014. Blood smears were air dried before fixation in ice cold methanol. Slides were then rehydrated in PBS and blocked in PBS with 5% skimmed milk before staining with monoclonal antibody against dsRNA (J2; 1:200) (Scicons, Hungary), washed and stained with secondary antibody; goat anti-mouse Alexa Fluor 595 (1:1000) from Molecular Probes (Life Technologies). The nuclei were stained with Hoechst 33342 (Life technologies). Coverslips were mounted with Fluoroshield (Sigma-Aldrich) and visualized on an inverted fluorescence microscope (Olympus IX81). (TIF) [file pone.0180293.s002.tif]

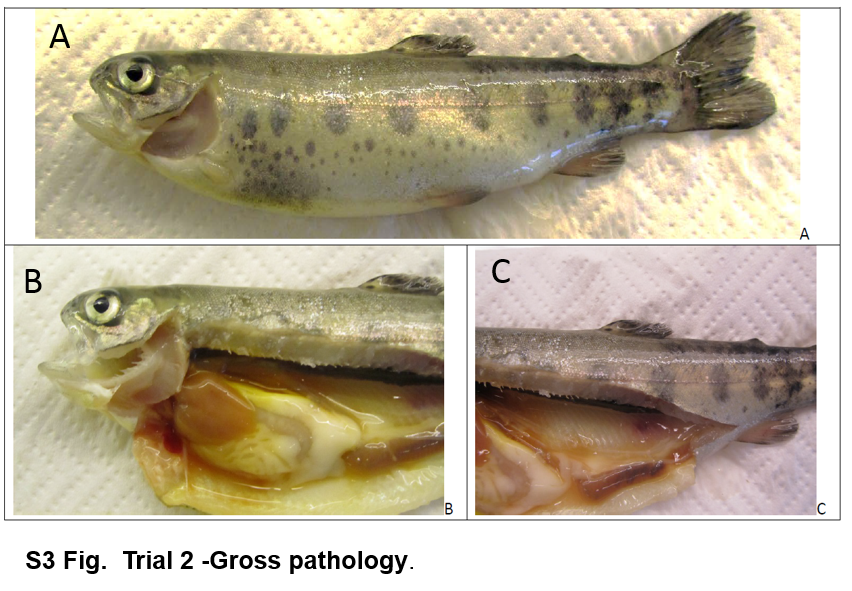

Supplement: S3 Fig — Injected rainbow trout 25 days post challenge. A) abdominal distension and pale gills. B) ascites and anaemia of internal organs. C) focal haemorrhage on abdominal wall. (TIF) [file pone.0180293.s003.tif]

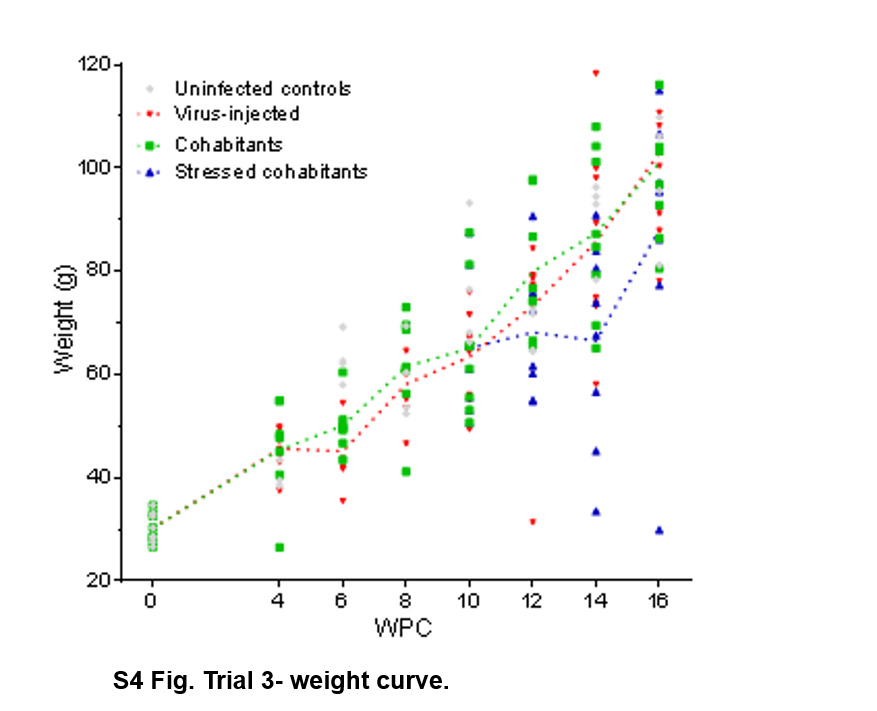

Supplement: S4 Fig — Weight curves based on the eight fish sampled per group at each time-point. Uninfected controls (grey), virus-injected fish (red), cohabitants (green) and stressed cohabitants (blue) are shown. Large dots represent individual measurements and the dotted trend lines show the group mean. The stressed cohabitants were exposed to an oxygen saturation of 40–50% for 4 h at 10 WPC and sampled at 12, 14 and 16 WPC. (TIF) [file pone.0180293.s004.tif]

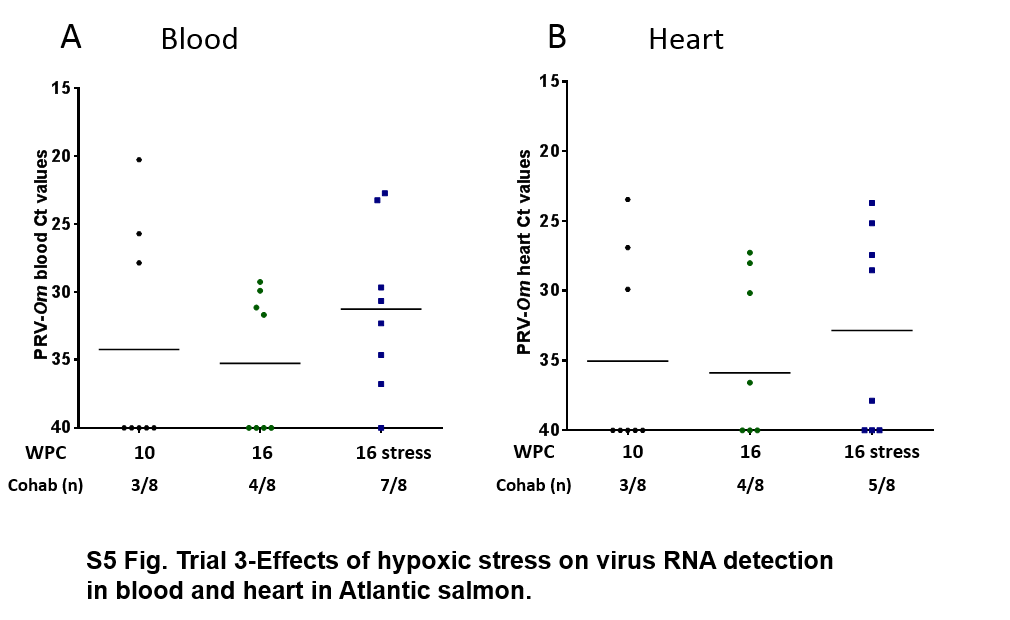

Supplement: S5 Fig — Virus analysis were performed by RT-qPCR targeting the PRV-Om segment S1 on blood (A) and heart (B) from cohabitants sampled at 10 WPC and cohabitants (open bars) and stressed cohabitants (blue bars) at 16 WPC/end of trial. The stressed cohabitants were exposed to an oxygen saturation of 40–50% for 4 h at 10 WPC. Dots represent individual Ct-values and the line show the group mean. The number of positive fish and total fish sampled are given below the figure (Cohab (n)). (TIF) [file pone.0180293.s005.tif]

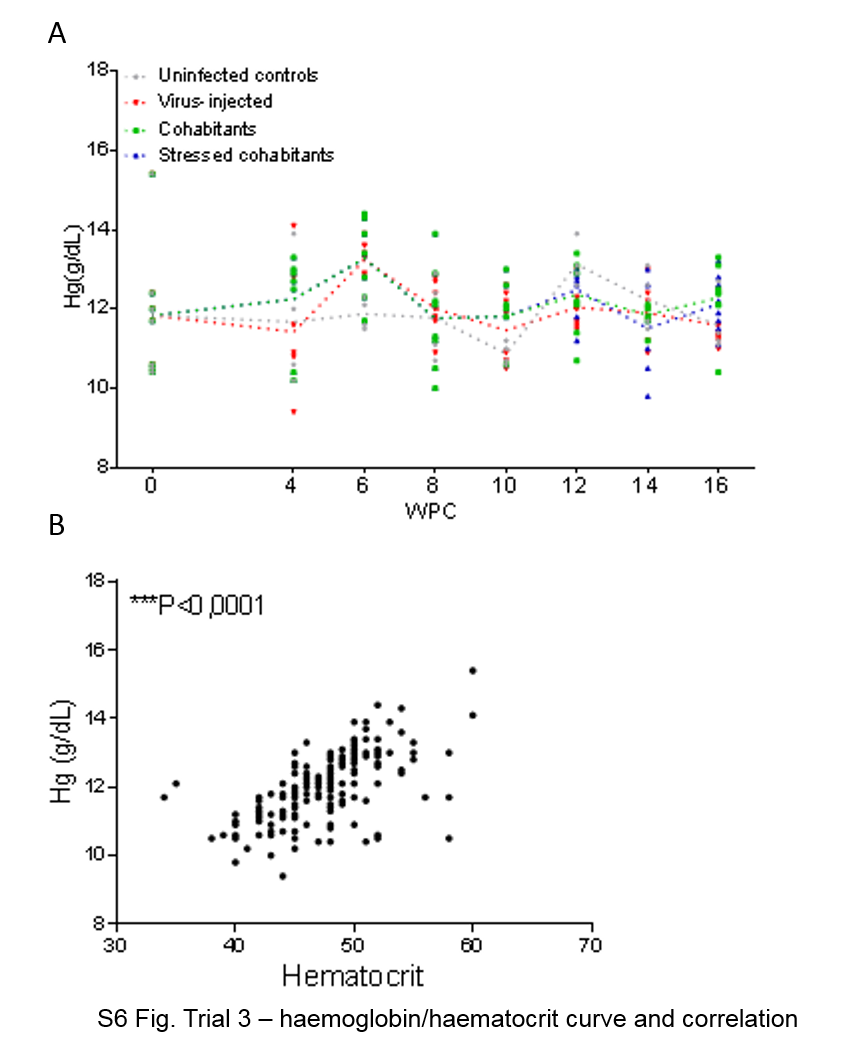

Supplement: S6 Fig — A) hemoglobin levels based on the eight fish sampled per group at each time-point. Uninfected controls (grey), virus-injected fish (red), cohabitants (green) and stressed cohabitants (blue) are shown. Large dots represent individual measurements and the dotted trend lines show the group mean. The stressed cohabitants were exposed to an oxygen saturation of 40–50% for 4 h at 10 WPC. The stress tested fish were sampled at 12, 14 and 16 WPC. Hemoglobin levels were measured using a HemoCue Hb 201+ with corresponding cuvettes, performed according to the manufacturers protocol (HemoCue). B) Correlation between hematocrit and hemoglobin levels measured in all groups. Hematocrit was measured using the standard procedure with micro haematocrit tubes (GMBH) and haematocrit centrifugation directly after blood sampling. (TIF) [file pone.0180293.s006.tif]
