# Supplementary material for: Infection experiments with novel Piscine orthoreovirus from rainbow trout (Oncorhynchus mykiss) in salmonids
Source: PLoS One. 2017 Jul 5;12(7):e0180293. doi: 10.1371/journal.pone.0180293 (PMC5497981; doi:10.1371/journal.pone.0180293)
Supplement: S1 Table — (DOCX) [file pone.0180293.s007.docx]

| Comparing Prevalence of PRV-Om positive Rainbow trout at 4 different time points in blood  and lesions in heart tissue. | **Statistically significant** |
| --- | --- |
|  | **NOT statistically significant** |

| **_4 WPC_** |  | _Positive_ | _Negative_ | _PREVALENCE_ |  |  | |  | _Positive_ | _Negative_ | _HISTOPATH_ |  |  |  |
| --- | --- | --- | --- | --- | --- | --- | --- | --- | --- | --- | --- | --- | --- | --- |
| **_SHEDDERS_** | _EXPOSED (tank 2)_ | _5_ | _0_ | _q-square_ | _10_ | _P value_ | | _0,0016_ | _4_ | _1_ | _q-square_ | _6,67_ | _P value_ | _0,0098_ |
|  | _NON EXPOSED (tank 4)_ | _0_ | _5_ | _q-square corrected YATES_ | _6,4_ | _P value_ | | _0,0114_ | _0_ | _5_ | _q-square corrected YATES_ | _3,75_ | _P value_ | _0,05_ |
|  | | | | | | | | | | | | | | |
| **_4 WPC_** |  | _Positive_ | _Negative_ |  |  |  | |  | _Positive_ | _Negative_ | _HISTOPATH_ |  |  |  |
| **_Cohabitants_** | _EXPOSED (tank 2)_ | _4_ | _1_ | _q-square_ | _6,67_ | _P value_ | | _0,0098_ | _0_ | _5_ | _q-square_ | _NA_ | _P value_ | _NA_ |
|  | _NON EXPOSED (tank 4)_ | _0_ | _5_ | _q-square corrected YATES_ | _3,75_ | _P value_ | | _0,05_ | _0_ | _5_ | _q-square corrected YATES_ | _NA_ | _P value_ | _NA_ |
|  | | | | | | | | | | | | | | |
| **_10 WPC_** |  | _Positive_ | _Negative_ |  |  |  | |  | _Positive_ | _Negative_ | _HISTOPATH_ |  |  |  |
| **_Shedders_** | _EXPOSED (tank 3)_ | _2_ | _3_ | _q-square_ | _2,5_ | _P value_ | | _0,1_ | _1_ | _4_ | _q-square_ | _1,11_ | _P value_ | _0,29_ |
|  | _NON EXPOSED (tank 4)_ | _0_ | _5_ | _q-square corrected YATES_ | _0,63_ | _P value_ | | _0,4_ | _0_ | _5_ | _q-square corrected YATES_ | _0_ | _P value_ | _1_ |
|  | | | | | | | | | | | | | | |
| **_10 WPC_** |  | _Positive_ | _Negative_ |  |  |  | |  | _Positive_ | _Negative_ | _HISTOPATH_ |  |  |  |
| **_Cohabitants_** | _EXPOSED (tank 3_ | _4_ | _1_ | _q-square_ | _6,67_ | _P value_ | | _0,0098_ | _4_ | _1_ | _q-square_ | _6,67_ | _P value_ | _0,0098_ |
|  | _NON EXPOSED (tank 4)_ | _0_ | _5_ | _q-square corrected YATES_ | _3,75_ | _P value_ | | _0,05_ | _0_ | _5_ | _q-square corrected YATES_ | _3,75_ | _P value_ | _0,05_ |
|  | | | | | | | | |  |  |  |  |  |  |
| **_12 WPC_** |  | _Positive_ | _Negative_ |  |  |  | |  | _Positive_ | _Negative_ | _HISTOPATH_ |  |  |  |
| **_Shedders_** | _EXPOSED (tank 2)_ | _2_ | _3_ | _q-square_ | _2,5_ | _P value_ | | _0,11_ | _1_ | _4_ | _q-square_ | _1,11_ | _P value_ | _0,29_ |
|  | _NON EXPOSED (tank 4)_ | _0_ | _5_ | _q-square corrected YATES_ | _0,63_ | _P value_ | | _0,4_ | _0_ | _5_ | _q-square corrected YATES_ | _0_ | _P value_ | _1_ |
|  | | | | | | | | | | | | | | |
| **_12 WPC_** |  | _Positive_ | _Negative_ |  |  |  | |  | _Positive_ | _Negative_ | _HISTOPATH_ |  |  |  |
| **_Cohabitants_** | _EXPOSED (tank 2)_ | _2_ | _3_ | _q-square_ | _2,5_ | _P value_ | | _0,11_ | _2_ | _3_ | _q-square_ | _1,11_ | _P value_ | _0,29_ |
|  | _NON EXPOSED (tank 4)_ | _0_ | _5_ | _q-square corrected YATES_ | _0,63_ | _P value_ | | _0,4_ | _0_ | _5_ | _q-square corrected YATES_ | _0_ | _P value_ | _1_ |
|  | | | | | | | | | | | | | | |
| **_14 WPC_** |  | _Positive_ | _Negative_ |  |  |  | |  | _Positive_ | _Negative_ | _HISTOPATH_ |  |  |  |
| **_Shedders_** | _EXPOSED (tank 4_ | _2_ | _3_ | _q-square_ | _2,5_ | _P value_ | | _0,11_ | _0_ | _5_ | _q-square_ | _NA_ | _P value_ | _NA_ |
|  | _NON EXPOSED (tank 4)_ | _0_ | _5_ | _q-square corrected YATES_ | _0,63_ | _P value_ | | _0,4_ | _0_ | _5_ | _q-square corrected YATES_ | _NA_ | _P value_ | _NA_ |
|  | | | | | | | | | | | | | | |
| **_14 WPC_** |  | _Positive_ | _Negative_ |  |  | |  |  | _Positive_ | _Negative_ | _HISTOPATH_ |  |  |  |
| **_Cohabitants_** | _EXPOSED (tank 4)_ | _3_ | _2_ | _q-square_ | _2,5_ | | _P value_ | _0,11_ | _4_ | _1_ | _q-square_ | _6,67_ | _P value_ | _0,0098_ |
|  | _NON EXPOSED (tank 4)_ | _0_ | _5_ | _q-square corrected YATES_ | _0,63_ | | _P value_ | _0,4_ | _0_ | _5_ | _q-square corrected YATES_ | _3,75_ | _P value_ | _0,05_ |
|  | | | | | | | | |  |  |  |  |  |  |
| **_All Experiment_** |  | _Positive_ | _Negative_ |  |  |  | |  | _Positive_ | _Negative_ | _HISTOPATH_ |  |  |  |
| **_Cohabitants_** | _EXPOSED_ | _13_ | _7_ | _q-square_ | _19,26_ | _P value_ | | _0_ | _9_ | _11_ | _q-square_ | _11,61_ | _P value_ | _0,0007_ |
|  | _NON EXPOSED_ | _0_ | _20_ | _q-square corrected YATES_ | _16,41_ | _P value_ | | _0,0001_ | _0_ | _20_ | _q-square corrected YATES_ | _9,18_ | _P value_ | _0,0025_ |
|  | | | | | | | | | | | | | | |
| **_All Experiment_** |  | _Positive_ | _Negative_ |  |  |  | |  | _Positive_ | _Negative_ | _HISTOPATH_ |  |  |  |
| **_Shedders_** | _EXPOSED_ | _11_ | _9_ | _q-square_ | _15,17_ | _P value_ | | _0,0001_ | _6_ | _14_ | _q-square_ | _7,06_ | _P value_ | _0,0079_ |
|  | _NON EXPOSED_ | _0_ | _20_ | _q-square corrected YATES_ | _12,54_ | _P value_ | | _0,0004_ | _0_ | _20_ | _q-square corrected YATES_ | _4,9_ | _P value_ | _0,026_ |
|  | | | | | | | | | | | | | | |
| **_All Experiment_** |  | _Positive_ | _Negative_ |  |  |  | |  | _Positive_ | _Negative_ | _HISTOPATH_ |  |  |  |
| **_All fish_** | _EXPOSED_ | _24_ | _16_ | _q-square_ | _19,5_ | _P value_ | | _0_ | _15_ | _25_ | _q-square_ | _18.46_ | _P value_ | _0_ |
|  | _NON EXPOSED_ | _0_ | _40_ | _q-square corrected YATES_ | _17,44_ | _P value_ | | _0_ | _0_ | _40_ | _q-square corrected YATES_ | _16.08_ | _P value_ | _0,0001_ |

Comparing Prevalence of PRV-Om positive Atlantic salmon in blood and lesions in heart tissue at 4 different time points.

| **_4 WPC_** | |  | _Positive_ | _Negative_ |  |  |  |  | _POS_ | _NEG_ | _HISTOPATH_ |  |  |  |
| --- | --- | --- | --- | --- | --- | --- | --- | --- | --- | --- | --- | --- | --- | --- |
| **_SHEDDERS_** | | _EXPOSED_ | _8_ | _0_ | _q-square_ | _12_ | _P value_ | _0,0005_ |  |  | _q-square_ | _Not Perf._ | _P value_ | _Not Perf._ |
|  |  | _NON EXPOSED_ | _0_ | _4_ | _q-square corrected YATES_ | _7,92_ | _P value_ | _0,004_ |  |  | _q-square corrected YATES_ | _Not Perf._ | _P value_ | _Not Perf._ |
|  | | | | | | | | | | | | | | |
| **_4 WPC_** | |  | _Positive_ | _Negative_ |  |  |  |  | _POS_ | _NEG_ | _HISTOPATH_ |  |  |  |
| **_Cohabitants_** | | _EXPOSED_ | _0_ | _8_ | _q-square_ | _NA_ | _P value_ | _NA_ |  |  | _q-square_ | _Not Perf._ | _P value_ | _Not Perf._ |
|  |  | _NON EXPOSED_ | _0_ | _4_ | _q-square corrected YATES_ | _NA_ | _P value_ | _NA_ |  |  | _q-square corrected YATES_ | _Not Perf._ | _P value_ | _Not Perf._ |
|  | | | | | | | | | | | | | | |
| **_6 WPC_** | |  | _Positive_ | _Negative_ |  |  |  |  | _POS_ | _NEG_ | _HISTOPATH_ |  |  |  |
| **_Shedders_** | | _EXPOSED_ | _8_ | _0_ | _q-square_ | _12_ | _P value_ | _0,0005_ |  |  | _q-square_ | _Not Perf._ | _P value_ | _Not Perf._ |
|  |  | _NON EXPOSED_ | _0_ | _4_ | _q-square corrected YATES_ | _7,92_ | _P value_ | _0,004_ |  |  | _q-square corrected YATES_ | _Not Perf._ | _P value_ | _Not Perf._ |
|  | | | | | | | | | | | | | | |
| **_6 WPC_** | |  | _Positive_ | _Negative_ |  |  |  |  | _POS_ | _NEG_ | _HISTOPATH_ |  |  |  |
| **_Cohabitants_** | | _EXPOSED_ | _0_ | _8_ | _q-square_ |  | _P value_ |  |  |  | _q-square_ | _Not Perf._ | _P value_ | _Not Perf._ |
|  |  | _NON EXPOSED_ | _0_ | _4_ | _q-square corrected YATES_ |  | _P value_ |  |  |  | _q-square corrected YATES_ | _Not Perf._ | _P value_ | _Not Perf._ |
|  | | | | | | | | | | | | | | |
| **_8 WPC_** | |  | _Positive_ | _Negative_ |  |  |  |  | _POS_ | _NEG_ | _HISTOPATH_ |  |  |  |
| **_Shedders_** | | _EXPOSED_ | _8_ | _0_ | _q-square_ | _12_ | _P value_ | _0,0005_ | _3_ | _5_ | _q-square_ | _2_ | _P value_ | _0,15_ |
|  |  | _NON EXPOSED_ | _0_ | _4_ | _q-square corrected YATES_ | _7,92_ | _P value_ | _0,004_ | _0_ | _4_ | _q-square corrected YATES_ | _0,5_ | _P value_ | _0,47_ |
|  | | | | | | | | | | | | | | |
| **_8 WPC_** | |  | _Positive_ | _Negative_ |  |  |  |  | _POS_ | _NEG_ | _HISTOPATH_ |  |  |  |
| **_Cohabitants_** | | _EXPOSED_ | _4_ | _4_ | _q-square_ | _3_ | _P value_ | _0,08_ | _0_ | _8_ | _q-square_ | _NA_ | _P value_ | _NA_ |
|  |  | _NON EXPOSED_ | _0_ | _4_ | _q-square corrected YATES_ | _1,17_ | _P value_ | _0,27_ | _0_ | _4_ | _q-square corrected YATES_ | _NA_ | _P value_ | _NA_ |
|  | | | | | | | | | | | | | | |
| **_10 WPC_** | |  | _Positive_ | _Negative_ |  |  |  |  | _POS_ | _NEG_ | _HISTOPATH_ |  |  |  |
| **_Shedders_** | | _EXPOSED_ | _8_ | _0_ | _q-square_ | _12_ | _P value_ | _0,0005_ | _4_ | _4_ | _q-square_ | _3_ | _P value_ | _0,083_ |
|  |  | _NON EXPOSED_ | _0_ | _4_ | _q-square corrected YATES_ | _7,92_ | _P value_ | _0,004_ | _0_ | _4_ | _q-square corrected YATES_ | _1,17_ | _P value_ | _0,279_ |
|  | | | | | | | | | | | | | | |
| **_10 WPC_** | |  | _Positive_ | _Negative_ |  |  |  |  | _POS_ | _NEG_ | _HISTOPATH_ |  |  |  |
| **_Cohabitants_** | | _EXPOSED_ | _3_ | _5_ | _q-square_ | _2_ | _P value_ | _0,15_ | _0_ | _8_ | _q-square_ | _NA_ | _P value_ | _NA_ |
|  |  | _NON EXPOSED_ | _0_ | _4_ | _q-square corrected YATES_ | _0,5_ | _P value_ | _0,47_ | _0_ | _4_ | _q-square corrected YATES_ | _NA_ | _P value_ | _NA_ |
|  | | | | | | | | | | | | | | |
| **_12 WPC_** | |  | _Positive_ | _Negative_ |  |  |  |  | _POS_ | _NEG_ | _HISTOPATH_ |  |  |  |
| **_Shedders_** | | _EXPOSED_ | _6_ | _2_ | _q-square_ | _6_ | _P value_ | _0,01_ | _3_ | _5_ | _q-square_ | _2_ | _P value_ | _0,15_ |
|  |  | _NON EXPOSED_ | _0_ | _4_ | _q-square corrected YATES_ | _3,38_ | _P value_ | _0,06_ | _0_ | _4_ | _q-square corrected YATES_ | _0,5_ | _P value_ | _0,47_ |
|  | | | | | | | | | | | | | | |
| **_12 WPC_** | |  | _Positive_ | _Negative_ |  |  |  |  | _POS_ | _NEG_ | _HISTOPATH_ |  |  |  |
| **_Cohabitants_** | | _EXPOSED_ | _3_ | _5_ | _q-square_ | _2_ | _P value_ | _0,15_ | _1_ | _7_ | _q-square_ | _0,55_ | _P value_ | _0,46_ |
|  |  | _NON EXPOSED_ | _0_ | _4_ | _q-square corrected YATES_ | _0,5_ | _P value_ | _0,47_ | _0+_ | _4_ | _q-square corrected YATES_ | _0,14_ | _P value_ | _0,719_ |
|  | | | | | | | | | | | | | | |
| **_14 WPC_** | |  | _Positive_ | _Negative_ |  |  |  |  | _POS_ | _NEG_ | _HISTOPATH_ |  |  |  |
| **_Shedders_** | | _EXPOSED_ | _5_ | _3_ | _q-square_ | _4,29_ | _P value_ | _0,03_ | _1_ | _7_ | _q-square_ | _0,55_ | _P value_ | _0,46_ |
|  |  | _NON EXPOSED_ | _0_ | _4_ | _q-square corrected YATES_ | _2,10_ | _P value_ | _0,143_ | _0+_ | _4_ | _q-square corrected YATES_ | _0,14_ | _P value_ | _0,719_ |
|  | | | | | | | | | | | | | | |
| **_14 WPC_** | |  | _Positive_ | _Negative_ |  |  |  |  | _POS_ | _NEG_ | _HISTOPATH_ |  |  |  |
| **_Cohabitants_** | | _EXPOSED_ | _3_ | _5_ | _q-square_ | _2_ | _P value_ | _0,15_ | _1_ | _7_ | _q-square_ | _0,55_ | _P value_ | _0,46_ |
|  |  | _NON EXPOSED_ | _0_ | _4_ | _q-square corrected YATES_ | _0,5_ | _P value_ | _0,47_ | _0+_ | _4_ | _q-square corrected YATES_ | _0,14_ | _P value_ | _0,719_ |
|  | | | | | | | | |  |  |  |  |  |  |
| **_16 WPC_** | |  | _Positive_ | _Negative_ |  |  |  |  | _POS_ | _NEG_ | _HISTOPATH_ |  |  |  |
| **_Shedders_** | | _EXPOSED_ | _4_ | _4_ | _q-square_ | _3_ | _P value_ | _0,083_ | _0_ | _8_ | _q-square_ | _NA_ | _P value_ | _NA_ |
|  |  | _NON EXPOSED_ | _0_ | _4_ | _q-square corrected YATES_ | _1,17_ | _P value_ | _0,279_ | _0_ | _4_ | _q-square corrected YATES_ | _NA_ | _P value_ | _NA_ |
|  | | | | | | | | | | | | | | |
| **_16 WPC_** |  | | _Positive_ | _Negative_ |  |  |  |  | _POS_ | _NEG_ | _HISTOPATH_ |  |  |  |
| **_Cohabitants_** | _EXPOSED_ | | _4_ | _4_ | _q-square_ | _3_ | _P value_ | _0,083_ | _3_ | _5_ | _q-square_ | _2_ | _P value_ | _0,15_ |
|  | _NON EXPOSED_ | | _0_ | _4_ | _q-square corrected YATES_ | _1,17_ | _P value_ | _0,279_ | _0_ | _4_ | _q-square corrected YATES_ | _0,5_ | _P value_ | _0,47_ |
|  | | | | | | | | | | | | | | |
| **_All time points analysed_** | | | _Positive_ | _Negative_ |  |  |  |  | _POS_ | _NEG_ | _HISTOPATH_ |  |  |  |
| **_Shedders_** | _EXPOSED_ | | _47_ | _9_ | _q-square_ | _53,35_ | _P value_ | _0_ | _11_ | _29_ | _q-square_ | _6,73_ | _P value_ | _0,0095_ |
|  | _NON EXPOSED_ | | _0_ | _28_ | _q-square corrected YATES_ | _50,00_ | _P value_ | _0_ | _0_ | _20_ | _q-square corrected YATES_ | _5,02_ | _P value_ | _0,025_ |
|  | | | | | | | | | | | | | | |
| **_All time points analysed_** | | | _Positive_ | _Negative_ |  |  |  |  | _POS_ | _NEG_ | _HISTOPATH_ |  |  |  |
| **_Cohabitants_** | _EXPOSED_ | | _17_ | _39_ | _q-square_ | _10,66_ | _P value_ | _0,0011_ | _5_ | _35_ | _q-square_ | _2,73_ | _P value_ | _0,098_ |
|  | _NON EXPOSED_ | | _0_ | _28_ | _q-square corrected YATES_ | _8,86_ | _P value_ | _0,0029_ | _0_ | _20_ | _q-square corrected YATES_ | _1,34_ | _P value_ | _0,247_ |
|  | | | | | | | | | | | | | | |
| **_All time points analysed_** | | | _Positive_ | _Negative_ |  |  |  |  | _POS_ | _NEG_ | _HISTOPATH_ |  |  |  |
| **_All fish_** | _EXPOSED_ | | _64_ | _48_ | _q-square_ | _29,47_ | _P value_ | _0_ | _16_ | _64_ | _q-square_ | _4,76_ | _P value_ | _0,029_ |
|  | _NON EXPOSED_ | | _0_ | _28_ | _q-square corrected YATES_ | _27,22_ | _P value_ | _0_ | _0_ | _20_ | _q-square corrected YATES_ | _3,39_ | _P value_ | _0,065_ |

| **Statistically significant** |
| --- |
| **NOT statistically significant** |
| **NA – not available** |
